# Comparative genomics of parasitic silkworm microsporidia reveal an association between genome expansion and host adaptation

**DOI:** 10.1186/1471-2164-14-186

**Published:** 2013-03-16

**Authors:** Guoqing Pan, Jinshan Xu, Tian Li, Qingyou Xia, Shao-Lun Liu, Guojie Zhang, Songgang Li, Chunfeng Li, Handeng Liu, Liu Yang, Tie Liu, Xi Zhang, Zhengli Wu, Wei Fan, Xiaoqun Dang, Heng Xiang, Meilin Tao, Yanhong Li, Junhua Hu, Zhi Li, Lipeng Lin, Jie Luo, Lina Geng, LinLing Wang, Mengxian Long, Yongji Wan, Ningjia He, Ze Zhang, Cheng Lu, Patrick J Keeling, Jun Wang, Zhonghuai Xiang, Zeyang Zhou

**Affiliations:** 1 State Key Laboratory of Silkworm Genome Biology, Southwest University, Chongqing, 400715, China; 2 College of Life Sciences, Chongqing Normal University, Chongqing, 400047, China; 3 Department of Botany, University of British Columbia, Vancouver, British Columbia, V6T 1Z4, Canada; 4 Beijing Genomics Institute at Shenzhen, Shenzhen, 518000, China

**Keywords:** Gene duplication, Horizontal gene transfer, Host-derived transposable element, Host adaptation, Microsporidian, Silkworms

## Abstract

**Background:**

Microsporidian *Nosema bombycis* has received much attention because the pébrine disease of domesticated silkworms results in great economic losses in the silkworm industry. So far, no effective treatment could be found for pébrine. Compared to other known *Nosema* parasites, *N. bombycis* can unusually parasitize a broad range of hosts. To gain some insights into the underlying genetic mechanism of pathological ability and host range expansion in this parasite, a comparative genomic approach is conducted. The genome of two *Nosema* parasites, *N. bombycis* and *N. antheraeae* (an obligatory parasite to undomesticated silkworms *Antheraea pernyi*), were sequenced and compared with their distantly related species, *N. ceranae* (an obligatory parasite to honey bees).

**Results:**

Our comparative genomics analysis show that the *N. bombycis* genome has greatly expanded due to the following three molecular mechanisms: 1) the proliferation of host-derived transposable elements, 2) the acquisition of many horizontally transferred genes from bacteria, and 3) the production of abundnant gene duplications. To our knowledge, duplicated genes derived not only from small-scale events (e.g., tandem duplications) but also from large-scale events (e.g., segmental duplications) have never been seen so abundant in any reported microsporidia genomes. Our relative dating analysis further indicated that these duplication events have arisen recently over very short evolutionary time. Furthermore, several duplicated genes involving in the cytotoxic metabolic pathway were found to undergo positive selection, suggestive of the role of duplicated genes on the adaptive evolution of pathogenic ability.

**Conclusions:**

Genome expansion is rarely considered as the evolutionary outcome acting on those highly reduced and compact parasitic microsporidian genomes. This study, for the first time, demonstrates that the parasitic genomes can expand, instead of shrink, through several common molecular mechanisms such as gene duplication, horizontal gene transfer, and transposable element expansion. We also showed that the duplicated genes can serve as raw materials for evolutionary innovations possibly contributing to the increase of pathologenic ability. Based on our research, we propose that duplicated genes of *N. bombycis* should be treated as primary targets for treatment designs against pébrine.

## Background

Microsporidia are obligate intracellular parasitic fungi that can infect a wide variety of organisms including vertebrate and invertebrate (particularly insects). Some species lead to severe syndromes in immunocompetent hosts and cause opportunistic infections in Acquired Immunodeficiency Syndrome (AIDS) patients [1,2]. More than 1200 microsporidia species that belong to 150 genera have been reported thus far [3]. Among them, the genus *Nosema* is the most diverse one. The domesticated silkworm, *Bombyx mori*, has long been considered as the primary source for the silk production worldwide. A highly mortal disease referred to as pébrine is currently the major threat to the silk production. Pébrine is caused by the infection of the microsporidian parasite, *Nosema bombycis*. This disease was first recognized during the destruction of the European silk industry in 1857 [4]. *N. bombycis* infects silkworms through vertical transmission from the mother host to their progenitive eggs, and chronically damages the entire body of the worm (including intestines, silk glands, muscles, and Malpighian tubules). After infections, the silkworm larvae are inactive and slow in development. Later, black spots, a disease symptom called pébrine [5], will appear throughout their bodies and lead to death. Since no effective treatment methods have been developed up to this point, the infections by *N. bombycis* inevitably cause devastating economic losses in the silkworm industry. Apart from the domesticated silkworms, *N. bombycis* can also infect various lepidopteran insects [6-8], indicative of their broad hosts range.

So far, the underlying genetic mechanisms of the highly infectious ability and the broad host range of *N. bombycis* remain unknown. To this end, we conducted a comparative genomic approach, from which we might learn a great deal about the genetic basis as to why and how *N. bombycis* can be so infectious across various hosts. In this study, we sequenced the genome of two microsporidian parasites: *N. bombycis* and *N. antheraeae* (an obligatory parasite to undomesticated silkworms *Antheraea pernyi*[9]). By comparing their genomes with a published distantly related *Nosema* genome, *N. ceranae*[10] (serving as outgroup), we show that the *N. bombycis* genome surprisingly expands due to the production of duplicated genes, the proliferation of host-derived transposable elements, and the acquisitions of many horizontally transferred genes from bacteria. Some duplicated genes associated with the cytotoxic pathway have experienced positive selection, implying that this adaptive evolution might enhance the infectious ability of *N. bombycis*, as well as the expansion of its host range. Considering that all reported microsporidian genomes are highly reduced and compact [11,12], our data, for the first time, reveal a usual genome evolution process showing that the genome of parasites could expand. Those expanded genetic gears might have influenced the infectivity and the survivorship of parasites as we report herein.

## Results

### Genomic architecture of *N. bombycis* and *N. antheraeae*

By using various sequencing platforms, 6.7X, 10X, and 28X physical coverage of whole genome sequence of *N. bombycis* were obtained from the Sanger sequencing method (plasmids with 2Kb inserts), the miniBAC end sequencing method, and the Illumina short-read sequencing method respectively (Additional file 1). Our sequencing efforts resulted in 1,605 scaffolds built from 3,551 contigs. The total assembly genome size is 15.7 Mb (the N50 of the scaffolds = 57.4 Kb and the maximum scaffold size = 571.1 Kb) (Additional file 2). A total of 4,458 protein coding sequences were identified (Table 1). The assembled genomic size (~15.7 Mb) is close to previous estimation by pulse-field gel electrophoresis (~15.3 Mb) [13], indicating that the coverage of the assembled genome is nearly complete. To sequence the genome of *N. antheraeae*, a total of 657 Mb of Illumina reads were obtained after filtering ambiguous reads. Our analysis results in 6,215 scaffolds and a total of 6.6 Mb of unique sequence. Totally, 3,413 protein coding sequences were identified (Table 1). The assembled genome size of *N. antheraeae* was estimated using the following equation: genome size = number of 15-mers per kilo-bases/depth of 15-mers per kilo-bases. The assembled genome size of *N. antheraeae* (~7.4 Mb) (Additional file 3) is close to our previous estimation by pulse-field gel electrophoresis [14], indicating that approximately 90% of the *N.antheraeae* genome was captured. A comparison of genome features among the generated *Nosema* genomes and published microsporidian genomes (one *Nosema* species and two *Encephalitozoon* species) is listed in Table 1.

**Table 1:** **A comparison of genome features among three *Nosema *species (*N. bombycis *, *N. antheraeae, and N. ceranae*) and two *Encephalitozoon *species (*E. cuniculi *and *E. bieneusi*)**

**Genomic features**	***N. bombycis***	***N. antheraeae***	***N. ceranae ***[10]	***E. cuniculi ***[24]	***E. bieneusi ***[26]
Chromosomes(bands)	18	~12	ND	11	6
Assembly(Mbp)	15.7	6.6	7.9	2.9	3.86
Genomic coverage	100%	~89%	90%	86%	~64%
Scaffold Num	1,605	6,215	5,465	11	1,646
N50(bp)	57,394	1,883	2,902	ND	2,349
Largest scaffold length(bp)	571,060	53,183	65,607	209,983	204,069
G + C content (%)	31	28	27	48	26
No .of CDS	4,458	3,413	2,614	1,997	3,632
Mean CDS length (bp)	741	775	904	1,077	995
GenBank No.	NA30919	NA183977	NA32971	NA155	NA21011

**Figure 1:**
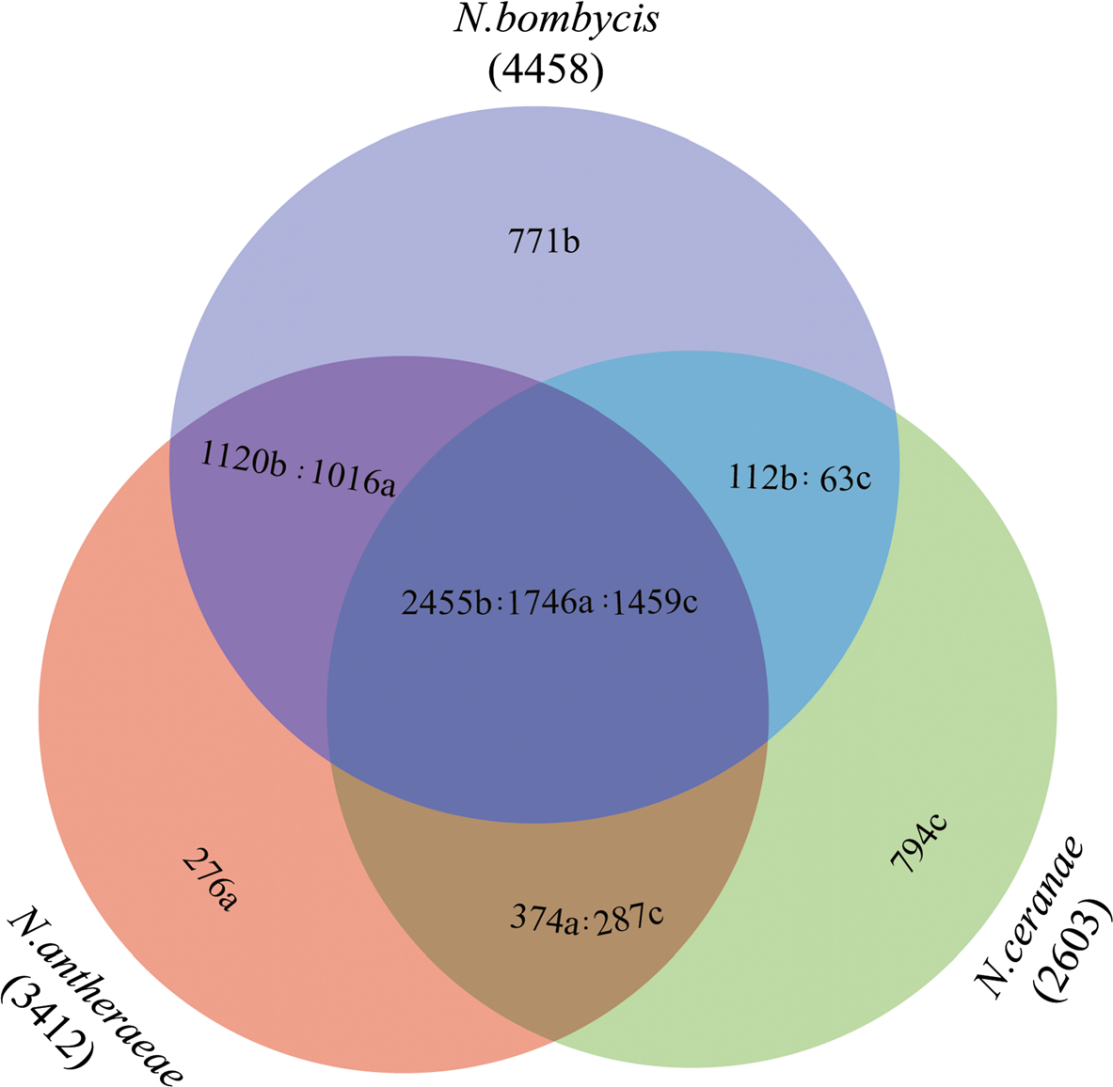
**Venn diagram showing the number of homologous genes and lineage-specific genes amongst three *Nosema *species, *N. bombycis*, *N. antheraeae*, and *N. ceranae*.** The arabic numbers followed by characters represent the number of homologous genes in each *Nosema* species (‘a’ denotes *N. antheraeae*, ‘b’ denotes *N. bombycis* , and ‘c’ denotes *N. ceranae*). For instance, 2455b:1746a:1459c means that 2455 genes of *N. bombycis*, 1746 genes of *N. antheraeae*, and 1459 genes of *N. ceranae* are homologous to each other.

To gain some insights into the variations of gene content among *N. antheraeae*, *N. bombycis*, and *N. ceranae*, the number of orthologous genes were compared. Most genes are shared among all three species, but 8% of genes were *N. antheraeae*-specific, 15.7% were *N. bombycis*-specific, and 30.5% were *N. ceranae-*specific (Figure 1). Gene ontology analysis revealed that no distinct differences were found among different gene functional categories (Additional file 4). Collectively, these three *Nosema* species lack genes for tricarboxylic acid cycle, oxidative phosphorylation, and fatty acid β-oxidation, consistent with previous observations [15-19]. Our observations further support that microsporidia do not possess tricarboxylic acid cycle and oxidative metabolism, and microsporidia parasites have often experienced on-going genome streamlining via the relaxation of purifying selection.

To determine if the *Nosema* proteins were more compact than other microsporidian parasites, the length of those silkworm *Nosema* proteins with assigned functions was compared to homologs of two published microsporidian parasitic *Encephalitozoon* species, *E. cuniculi* and *E.intestinalis* (Additional file 5). Our results show that the average length of total homologous genes from *N. antheraeae* and *N. bombycis* is shorter than that from *E. cuniculi* and *E. intestinalis*, indicating that proteins in *Nosema* were more compact than those in *Encephalitozoon*.

Overall, our comparative genomics analysis showed that *N. bombycis* possesses a much larger genome size than other two *Nosema* species (Table 1). Considering that *N. bombycis* has wide host range, the genome expansion might facilitate the host adaption in *N. bombycis*. Thus, for the subsequent analyses, we aim to seek for the underlying genetic mechanisms as to why and how *N. bombycis* genome expands. Furthermore, we seek for the putative genetic components that contribute to the infectious ability of *N. bombycis* in a hope that our analyses could provide some clues on the development of treatment strategies of pébrine.

### Proliferation of host-derived transposable elements in *N. bombycis*

After obtaining the genomes of the two *Nosema* species, we seek for the potential molecular mechanisms underlying the genome expansion of *N. bombycis*. Considering that the proliferation of transposable elements often contributes to the genome size variation in many eukaryotes [20], it was considered as the first molecular mechanisms for us to check. Although the genomes of several human pathogenic microsporidians have been shown to lack transposable elements, transposable elements have been detected in the genomes of other non-human pathogenic microsporidians [21-25]. To understand what degree those transposable element shape the genomic architectures in *Nosema*, we searched for transposons in *N. bombycis* and *N. antheraeae* (for details, see Materials and Methods). Two different approaches were implemented in this study. Because most transposable elements comprise internal protein-coding genes (e.g., transposase or reverse transcriptase) that are necessary for their transposition, we first identified those putative transposable elements by searching for their internal protein-coding sequences. In many cases, the internal protein-coding sequences are highly generated but recognizable. Second, for those that do not possess readily identifiable internal protein-coding sequences, other features such as inverted repeats or insertion sites were used to recognize the transposable elements.

**Figure 2:**
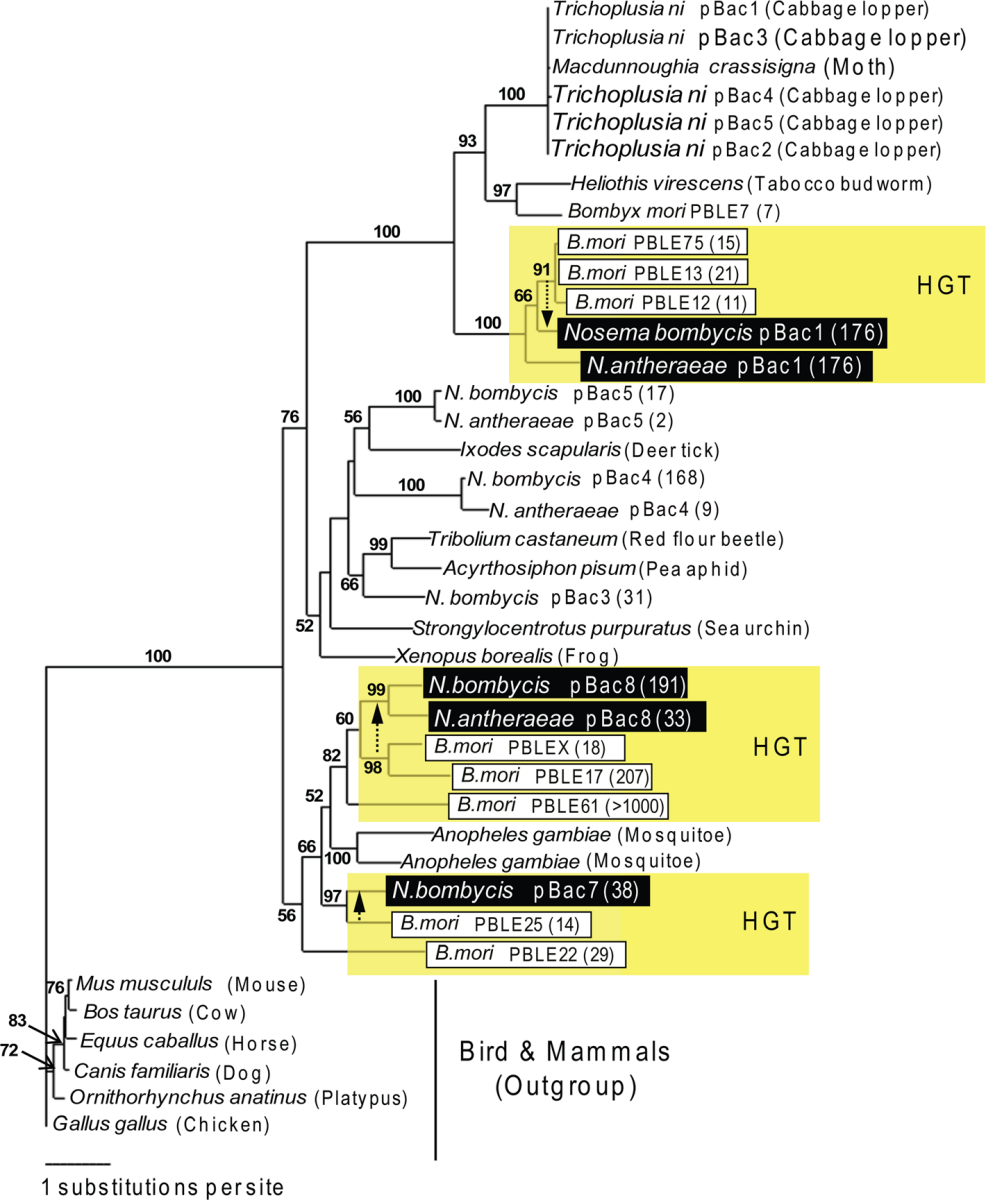
**A maximum-likelihood phylogenetic tree of host-derived *Piggybac *transposase sequences.** Arrows show the putative recent horizontal gene transfer (HGT) events of host-derived transposable elements. Several transposons are closely related to those from insects. Black boxes indicate elements from the two silkworm-infecting *Nosema* species, while white boxes indicate elements from the domesticated silkworm *B. mori*. Numbers in parentheses indicate the total copy numbers for each transposable element.

Overall, our results show evidence that a larger genome size of *N. bombycis* is partly due to both the acquisition of new transposons and expansion of existing transposable elements (Table 2). Among all identified transposable elements, the *Ty3*/*gypsy* retrotransposons [22] constitute the largest part of known classes of transposable elements in *N. bombycis*. A broad sampling from GenBank shows that these transposable elements also reside in other microsporidian groups including *Spraguea lophii*, *Edhazardia aedis*, and *Brachiola algerae*, indicating that this transposable element family exists back to the common ancestor of most microsporidian species and further expand in *N. bombycis*. Majority of transposable elements among *Nosema* genomes are common across three *Nosema* species, whereas *Piggybac* transposons were only found in *N. antheraeae* and *N. bombycis* except for *N. ceranae* (Additional file 6). To test whether *Piggybac* was lost during the evolution of *N. ceranae* or was gained in the most recent common ancestor of *N. antheraeae* and *N. bombycis*, the phylogeny of *Piggybac* was reconstructed from *Nosema*, domesticated silkworms, and other insects. Our analyses show that the *Nosema Piggybac* sequences fall into four well-supported groups, and three out of them are closely related to *Piggybac* elements from domesticated silkworms (Figure 2). Although the exact relationships of these *Piggybac* elements between *Nosema* and *Bombyx* is complicated, our phylogenetic analysis suggests that *Piggybac* was acquired in the most common ancestor of *N. antheraeae* and *N. bombycis* through horizontal transfer events from possibly host silkworms. These transfers likely independently took place three times, leading to the three major subgroups (labeled as HGT in Figure 2). In addition, the *Nosema pBac3,4,5* are closely related to the elements from other insects (in the middle part of tree; Figure 2), suggesting that these *Nosema Piggybac* elements might originate from the insects. To rule out the possibility of the host contaminations during the genome assembly, we amplified regions flanking *Piggybac* elements using *Nosema*-specific primers, and confirmed the existence of *Piggybac* in *Nosema* genomes (data not shown).

**Table 2:** **Classification of repetitive families in *N. bombycis *genome**

**Type**	**Subtype**	**Length (bp)**	**Percent (%) of genome**
DNA	hAT	1,011,459	6.45
	Merlin	470,573	3.00
	PiggyBac	441,876	2.82
	TcMar	786,733	5.02
	MuDR	109,681	0.70
	others	58,866	0.38
LTR	Gypsy	577,653	3.68
	others	33,635	0.21
LINE	Dong-R4	162,622	1.04
	others	59,703	0.38
Rolling-circle	Helitron	102,334	0.65
SINE	—	28,669	0.18
Unknown	—	2,204,497	14.06
Total	—	6,048,301	38.57

Since the host-derived *Piggybac* elements are so abundant in *N. bombycis*, can those host-derived *Piggybac* elements serve as the vector of capturing host-derived genes? To answer this question, we checked the host-derived *Piggybac* elements that do not have any readily identifiable internal protein-coding sequences. Because they are usually hard to be identified due to the lack of the internal readily recognizable protein-coding genes, the terminal inverted repeat (ITR) and the insertion site (TTAA) of the *Piggybac* elements were used as the criteria for our search. In other words, we searched for the *N. bombycis* genomic regions that are flanked by the ITR and the insertion site (TTAA) of the *Piggybac* elements and comprise “*extrinsic*” sequences. After identification of those *Piggybac* elements, we examine whether the “*extrinsic*” sequences were recently transferred by the transposition of the *Piggybac* elements that are specific to *N. bombycis* via comparing the colinearity of these regions with those in *N. antheraeae* and *N. ceranae*. A total of 17 *Piggybac* elements with an internal “*extrinsic*” sequence were identified *(*Additional file 7). Among them, only one case might be recently gained in *N. bombycis* based on the colinearity (Additional file 8 and Additional file 9). When we blasted the internal “*extrinsic*” sequence of the 17 *Piggybac* elements in GenBank using the “nr” database by the blastn function, no detectable similarity with any known sequences was found. Our analysis thus far suggests that the host-derived *Piggybac* elements might not be able to serve as the vector of capturing genes from hosts to *N. bombycis*.

### Horizontally transferred protein-coding genes is another source of genetic expansion in *N. bombycis*

**Figure 3:**
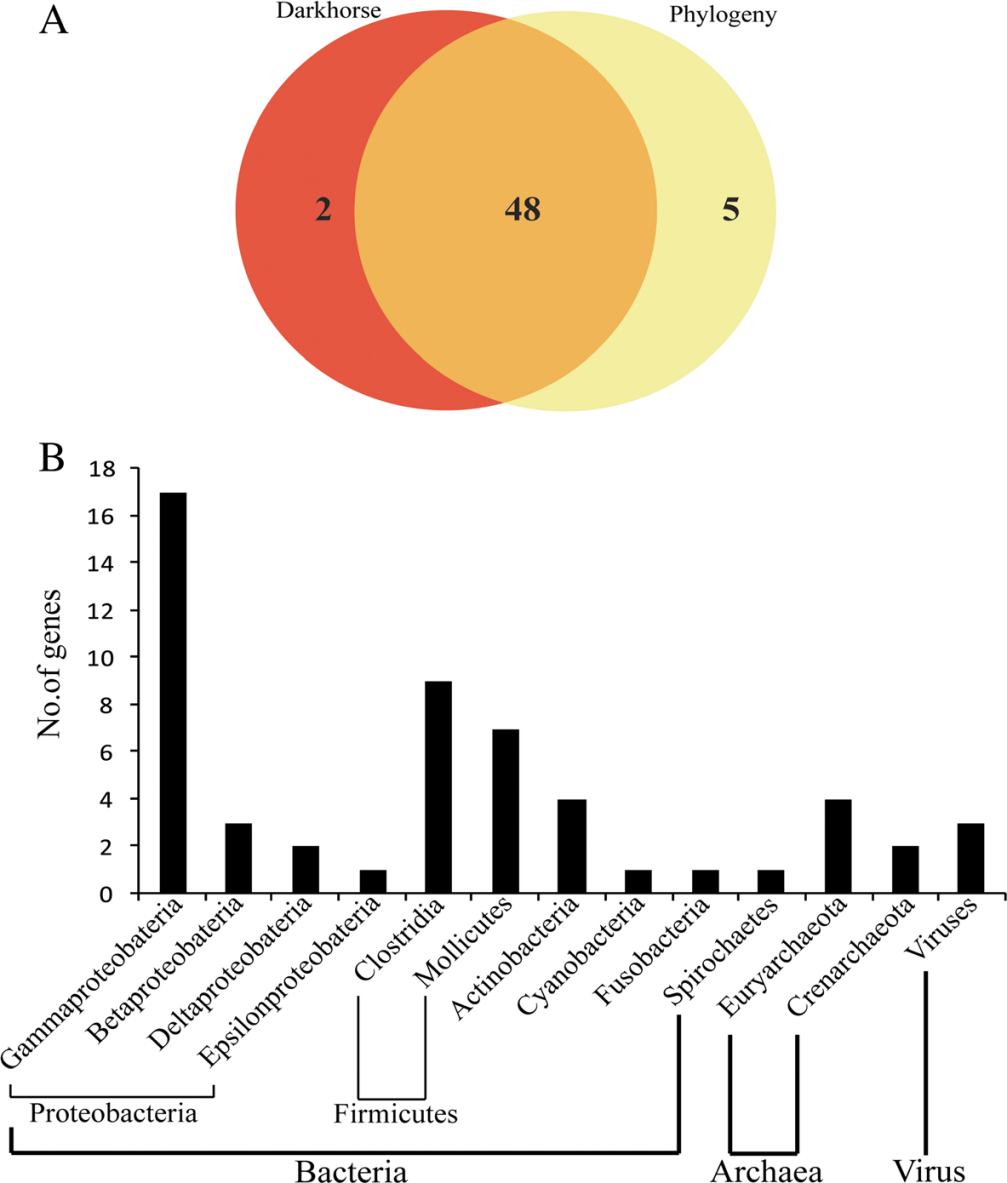
**Horizontal gene transfers of protein-coding genes in *N. bombycis*.** (**A**) Venn diagram showing the numbers of HGT genes between two different dataset that were identified using two different methods, the Darkhorse method and the phylogenetic method. The total number of the union of HGT genes between two dataset is 55. (**B**) The diagram showing the origination of those 55 HGT genes. All of them originated from prokaryotes.

Since *N. bombycis* has experienced the proliferation of native and host-derived transposons, we sought to determine if horizotally transferred protein coding genes from other organisms can also facilitate the genome expansion in *N. bombycis*, as was recently found in *Encephalitozoon*[26,27]. To maximize the likelihood of detecting horizontal protein coding gene transfer (HGT) events in *N. bombycis*, we implemented two different approaches: a genome-wide prediction method based on orthologous sequences using the software Darkhorse, and a phylogenetic method where we screened the putative HGTs in a total of 4458 gene family phylogenies. Overall, these two different approaches identify 50 and 53 different HGTs in *N. bombycis*, respectively. Among them, 48 genes are shared between these two approaches (Figure 3A), resulting in a set of 55 union HGT genes between two different dataset. By investigating the taxonomic origin of these 55 unions HGT genes in a phylogenetic framework, all of them were transferred from prokaryotes (Figure 3B). No host-derived genes were found in our analysis, further suggesting that only host-derived transposable elements can be transferred into the *N. bombycis* genome instead of host-derived protein-coding genes. Using the clusters of orthologous group database, we found that 21 HGT candidates are unknown in functions, and 34 are predicted to fall into diverse gene functions (Additional file 10). Among 34 HGT genes, five genes are involved in nucleotide metabolism and two genes are involved in sugar metabolism. Interestingly, one HGT gene that was annotated as phosphomevalonate kinase (EC2.7.4.2) is shown to be an important player in the mevalonate pathway of *N.bombycis* (Additional file 11). In the mevalonate pathway, phosphomevalonate kinase is a key enzyme to catalyze the rate-limiting step for the production of isopentenyl pyrophosphate (IPP). IPP is important for various molecular functions such as terpenoid synthesis, protein prenylation, cell membrane maintenance, protein anchoring, and N-glycosylation. Overall, our observations lead us to hypothesize that some of HGTs might play a pivotal role on the adaptation or survivorship of *N. bombycis* over the course of evolution. Alternatively, many HGTs might be merely neutral without any immediate adaptive consequences after their transfers. Further hypothesis testing will be necessary.

### Recent gene duplication events contribute to the genome expansion in *N. bombycis*

**Figure 4:**
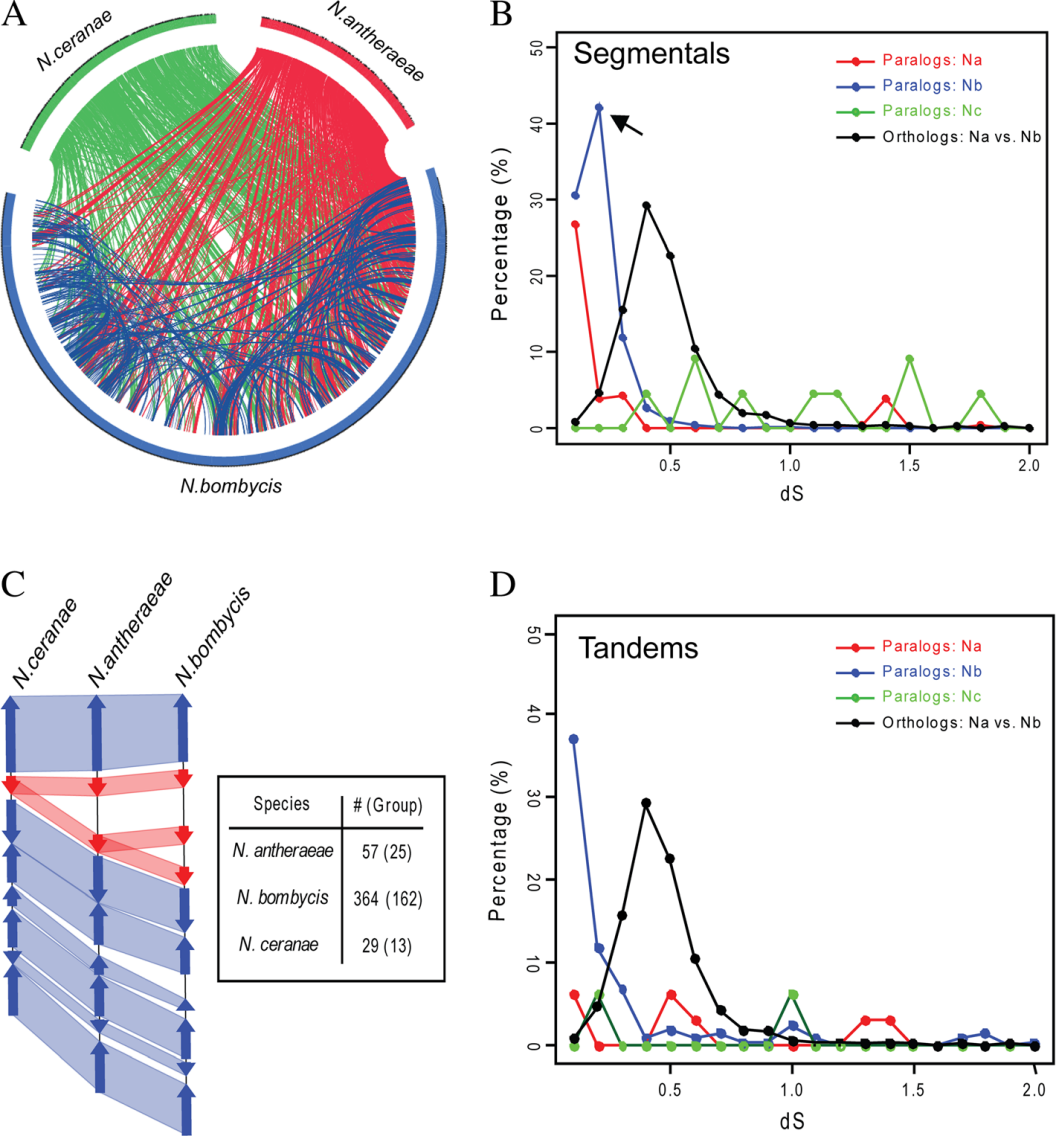
**Gene duplications and the dS distribution of paralogs and orthologs among three *Nosema *species.** Abbreviation: Na, *N. antheraeae*; Nb, *N. bombycis*; Nc, *N. ceranae*. (**A**) A circos map showing the comparative genomics among three different *Nosema* species based on all available scaffolds. Each line represents the homologous syntenic regions between any two species or between any given two chromosome positions of single species. Many lines across different scaffolds of *N. bombysis* indicates higher rate of segmental syntenic duplications. (**B**) The dS distribution of segmental paralogs of Nb and the orthologs between Nb and Na showing a higher dS values in orthologs in general. Notably, a higher peak (arrow) seen in Nb suggests the possibility of a burst of paralogs recently over the Nb evolution after the separation of Na and Nb. (**C**) An example of syntenic comparisons among three *Nosema* species showing a cluster of tandem paralogs. The corresponding genetic position and names of identified element are provided in Additional file 8. The number of all identified tandem paralogs for each *Nosema* genome is summarized on the right side. (**D**) The dS distribution of tandem paralogs of Nb and orthologs between Na and Nb showing that majorities of tandem paralogs arose after the separation of Na and Nb because the dS values of those tandem paralogs are smaller than that of orthologs.

**Figure 5:**
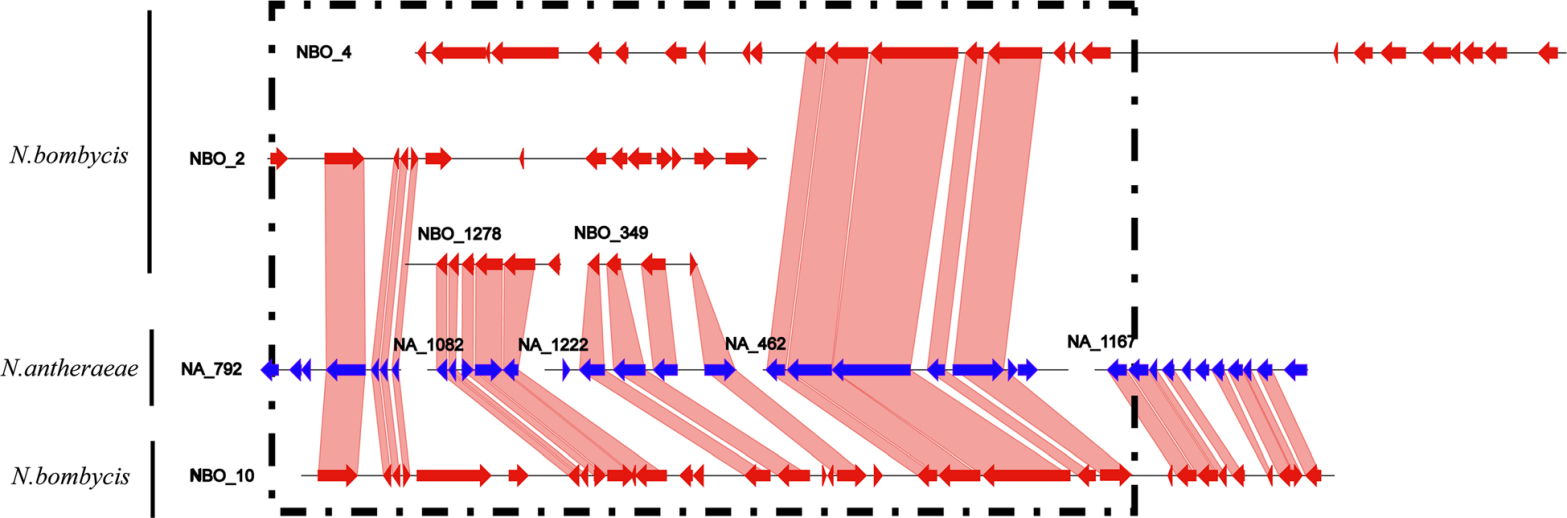
**An example from the syntenic analysis showing that *N. bombycis *often consist of two homologous regions, instead of one such as *N. antheraeae*.** When summarized the number of syntenic regions in both *N. antheraeae* and *N. bombycis*, the number of paralogous syntenic region of *N. bombycis* is often twice more than that of orthologous syntenic region between *N. antheraeae* and *N. bombycis*, indicating that large segmental duplication events have occurred over the evolution of *N. bombycis*.

Although our previous analyses showed that the proliferation of host-derived transposable elements and horizontally transferred genes could contribute to the genome size expansion in *N. bombycis*, their contributions are not sufficient to explain the much larger genome size of *N. bombycis* than other two small-genome *Nosema* species (*N. antheraeae* and *N. ceranae)*. Considering that gene duplication is a common molecular mechanism mediating the expansion of genome size in many eukaryotes [28], we then seek for the evidence if gene duplications also play a role on the genome expansion in *N. bombycis*. We first performed a syntenic analysis to identify possible segmental duplication events in each *Nosema* species. Among three species, we found that *N. bombycis* contained 942 pairs of segmental duplications throughout its genome (Figure 4A, Additional file 12). In contrast, almost no segmental duplication could be detected in either *N. antheraeae* or *N. ceranae*. Because the assemblies of all these genomes are fragmented, it is not possible to conclude whether these segmental duplications are large in number and spread throughout the genome, or arise due to multiple whole chromosome duplication events or an ancient whole genome duplication event. Nevertheless, we have identified a region where it appears that a single large-scale duplication event explain the data better than several independent large-scale duplication events (Figure 5). To date these duplication events, we estimated synonymous substitution rate (*dS*) for paralogous genes from segmental duplications in *N. bombycis*, and compared them with the *dS* derived from orthologs between *N. antheraeae* and *N. bombycis*. The *dS* values are commonly used as the proxy of age of gene duplication because the synonymous substitutions evolve in a neutral fashion [28]. On average, the *dS* values of paralogs from segmental duplications in *N. bombycis* are generally lower than that of orthologs between *N. antheraeae* and *N. bombycis* (Figure 4B), suggesting that these duplication events took place after the separation of *N. antheraeae* and *N. bombycis*. In addition to the detection of segmental duplications, we identified numerous tandem duplication events among three *Nosema* species. We detected a higher rate of tandem duplications in *N. bombycis* compared to other two *Nosema* species, and in some cases multiple events could be mapped at a single locus (Figure 4C). On average, the *dS* values of these paralogs are also much lower than that of orthologus genes between *N. bombycis* and *N. antheraeae* (Figure 4D), indicating that most tandem paralogs in *N. bombycis* also arose relatively recent after the separation between *N. bombycis* and *N. antheraeae*. In short, the *N. bombycis* genome has expanded in size largely due to many large-scale and small-scale gene duplication events.

### Adaptive evolution of duplicated genes might enhance the pathogenic ability in *N. bombycis*

Paralogs often provide raw materials for evolutionary innovations, including the survival of parasites in their hosts [29]. We therefore sought to identify possible instances of adaptive changes associated with the pathogenic ability of *N. bombycis* among those duplicated genes derived from large-scale duplication events in *N. bombycis*. First, we examine if paralogs of *N. bombycis* contribute to the adaptive evolution more often than orthologs among all *Nosema* species. Clusters of homologous genes in *N. bombycis* were classified to four different groups: 1) clusters of orthologus genes (COGs) of 1:1:1 trios of *N. bombycis*, *N. antheraeae*, and *N. ceranae*, 2) COGs of 1:1 gene pairs of *N. bombycis* and *N. antheraeae*, 3) COGs of 1:1 gene pairs of *N. bombycis* and *N. ceranae*, and 4) clusters of paralogous genes (CPGs) in *N. bombycis*. Pairwise *dN*/*dS* ratio analyses for these four different clusters of homologous genes were computed and their cumulative *dN*/*dS* ratio curve were compared (see Materials and Methods for details). Compared to COGs, a higher proportion of CPGs in *N. bombycis* showed higher value of *dN*/*dS* ratio, suggesting that CPGs are evolving at a faster rate than COGs at the amino acid level (Additional file 13). In most cases, this is likely due to the relaxation of purifying selection. However, we observed that a higher proportion of CPGs showed *dN*/*dS* ration greater than 1, indicative of positive selection. Overall, our observations support the view that CPGs contributed more to adaptive evolution than COGs in *N. bombycis*.

**Figure 6:**
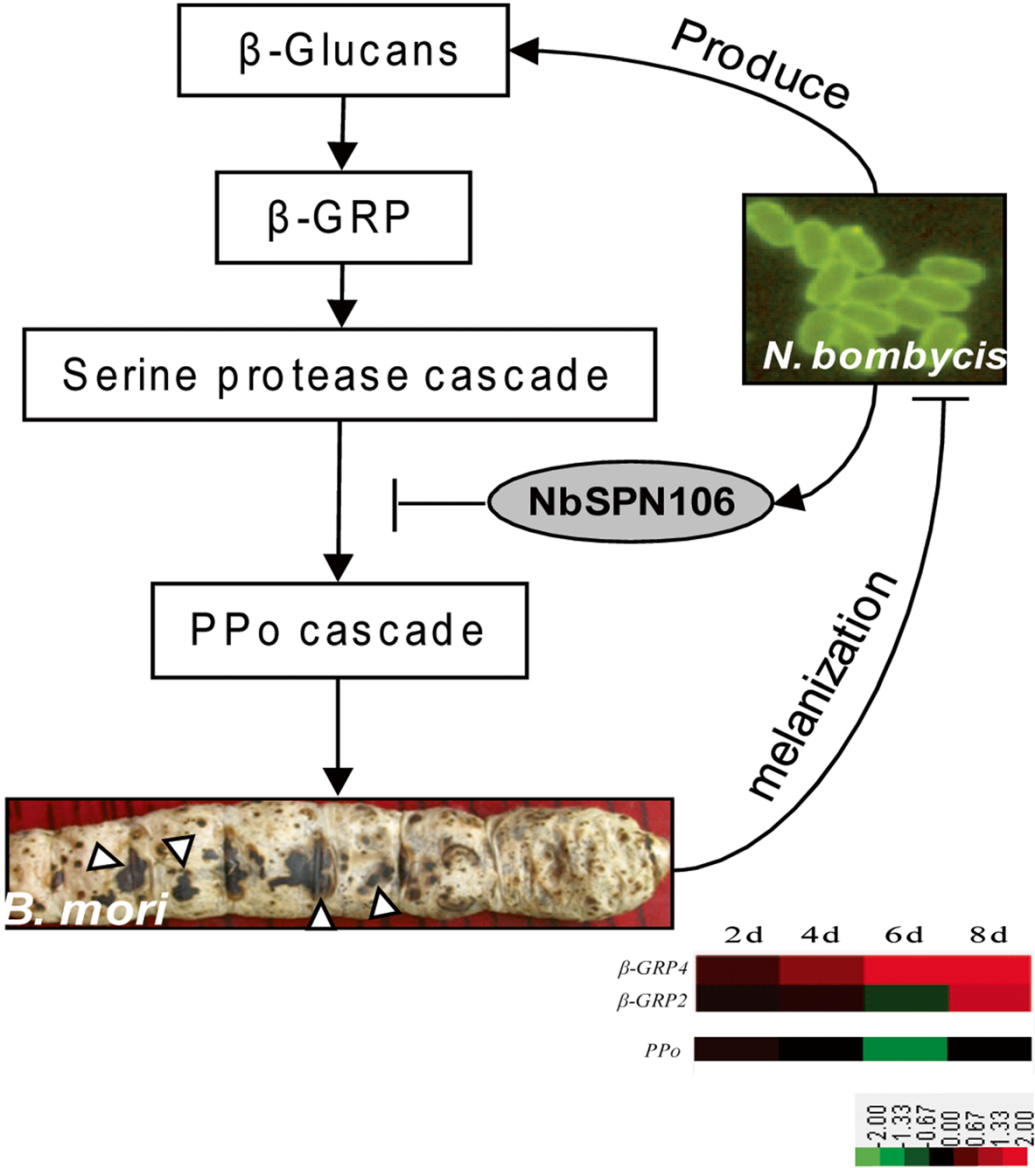
**A hypothetical model showing how the SPN protein of *N. bombycis *suppresses the serine protease cascade of the melanization pathway of the host *B. mori*.** After the suppression of the serine protease cascade, the defensive response, the subsequent formation of melanization will be inhibited in the hosts. Abbreviation: PPO, prophenoloxidase; β-GRP, β-glucan recognition protein.

To examine if any particular codons of CPGs of *N. bombycis* have undergone positive selection, we applied a site model approach with maximum likelihood using the software PAML (see Materials and Methods for details). The results show that 24 out of 240 CPGs have experienced positive selection in *N. bombycis* (Table 3), and 62% (37/60) genes in 24 CPGs have the support of EST tags (Additional file 14). The estimated parameters and positively selected sites for those positively selected CPGs are shown in Additional file 15. Although the majority of positively selected CPGs are hypothetical proteins with unknown functions, a handful of them are not. For example, CPG844 is related to LPXTG motif cell wall anchor domain protein and CPG1776 is related to surface adhesion protein. Positive selection acting on these two CPGs might play an important role in host recognition and interaction since they are involved in surface adhesion. Other examples are two positively selected CPGs that are related to serine protease inhibitor (SPN106). The functions of serine protease inhibitor have been shown to decrease the immune responses in hosts [30-32]. In the melanization pathway of *B. mori*, serine protease cascade is one of the most important biochemical reactions to inhibit the propagation of pathogens [33]. We scanned the gene expression pattern of *B. mori* post infection of *N. bombycis* by microarray analysis and found that the key gene PPO in melanization pathway was significantly suppressed. From our gene chip expression analysis (unpublished data), we found that the gene expression of two upstream regulators of host PPO melanization pathway, *β-GRP2* and *β-GRP4*, in *B. mori* are up-regulated during the infection of *N. bombycis* (Figure 6). The upregulation of *β-GRP2* and *β-GRP4* subsequently suppress the production of PPO against the infection of *N. bombycis*. To seek for the treatment method of pébrine, the interplay between SPN106 and *β-GRP* should be treated as our priority in the future studies. These observations lead us to hypothesize that adaptive evolution of serine protease inhibitor in *N. bombycis* might deter the melanization pathway by blocking the serine protease cascade in domesticated silkworms (Figure 6).

**Table 3:** **Site test of adaptive evolution for *N. bombycis *paralogous genes**

**Description**	**CPG**	**Members**	**Duplicate type**	**LRT statistics**			**dN/dS**
				**M1 vs. M2**	**M7 vs. M8**	**M8 vs. M8a**	
Hypothetical protein	CPG199	4	DD	17.30***	17.68***	17.07***	6.55472
Hypothetical protein	CPG293	4	SD,DD	7.34*	7.58*	6.68*	67.43545
Hypothetical protein	CPG446	3	TD	15.34***	16.15***	16.52***	96.64162
Hypothetical protein	CPG767	3	TD,DD	13.80**	13.92***	13.26**	12.30571
Surface adhesion protein	CPG844	3	SD, DD	21.04***	21.03***	22.81***	6.72603
Serine protease inhibitor 106	CPG945	3	TD,DD	5.85	5.92	5.8	19.13308
Serine protease inhibitor 106	CPG1974	2	DD	15.81***	16.31**	15.81***	52.1588
LPXTG-motif cell wall anchor domain protein+	CPG1776	2	SD	14.38***	14.39**	14.38***	20.02006
DnaJ homolog subfamily C member 9	CPG1792	2	SD	13.76**	13.85***	19.87***	57.65414
NIK and IKK(beta) binding protein	CPG1878	2	SD	12.22**	12.59**	14.57***	36.70677
Replication-associated protein	CPG2140	2	DD	18.67***	18.76***	21.14***	15.15205
Glucan endo-1,6-beta-glucosidase	CPG1640	2	DD	5.72	5.72	9.64**	61.53906
Integrator complex subunit 4	CPG2120	2	SD	8.46*	8.59*	8.22*	14.58649
Hypothetical protein	CPG961	3	DD	21.04***	22.99***	19.99***	9.5982
Hypothetical protein	CPG1520	2	SD	15.28***	15.37***	15.13***	8.84271
Hypothetical protein	CPG1637	2	SD	18.44***	18.75***	20.57***	14.62068
Hypothetical protein	CPG987	3	DD	13.65**	13.37**	13.11**	39.08442
Hypothetical protein	CPG1865	2	SD	10.90**	10.94**	10.70**	23.75418
Hypothetical protein	CPG1967	2	SD	6.06*	6.81*	5.48	29.25563
Hypothetical protein	CPG2026	2	DD	8.28*	8.41*	15.98***	15.9724
Hypothetical protein	CPG2083	2	DD	29.01***	29.00***	31.16***	61.60344
hypothetical protein	CPG775	3	SD	14.05***	14.05***	12.37**	5.3171
Hypothetical protein	CPG2128	2	SD	17.79***	17.85***	19.27***	45.49562
Hypothetical protein	CPG869	3	SD, DD	17.45***	17.85***	17.31***	8.16665

Note: SD, segmental; TD, Tandem; DD, Disperse; M1 vs. M2, LRT statistic for model M1 versus M2; M7 vs. M8, LRT for model M7 versus M8; * Significance with P < 0.05; ** Significance with P < 0.01; *** Significance with P < 0.001.

## Discussion and conclusion

Preventing the infection of *Nosema bombycis* is one of the prime concerns in the domesticated silkworm industry. However, attempts to manage these pathogens have been hindered by our poor knowledge of the underlying molecular mechanisms contributing to the highly infectious ability of *N. bombycis*. In the absences of transformation, genetics, and axenic cultivation, the comparative genomics approach is one of the few tools available to tackle these issues. In this study, we compared the genome of the major commercial silkworm pathogen, *N. bombycis*, to those of *N. antheraeae* and *N. ceranae*. Our study showed that the large genome size in silkworm *Nosema* genome is due to the proliferation of host-derived transposable elements, horizontally transferred genes from prokaryotes, and the production of segmental and tandem duplicates. Previous studies on the characterization of microsporidian genomic architectures have focused more on the genome reduction aspect [15-19,34-36]. Although few studies assumed the possibility of genome expansion in the microsporidia [23,37], the direct evidence is lacking. From the genome streamlining perspective, it is evident that many metabolic essential genes (e.g., the tricarboxylic acid cycle, fatty acid β-oxidation, respiratory electron-transport chain always) are missing in the microsporidian genome. In stark contrast, our study provides the first solid evidence showing that the microsporidian genome can expand. Gene duplications and proliferation of host-derived transposable elements are the two predominant molecular mechanisms contributing to the genome expansion in *N. bombycis*.

Recently, two studies have reported that some genes in the microsporidia *Encephalitozoon romaleae* were derived from an ancestral host [38,39], but we did not find any evidence of host-derived genes in *N. bombycis*. Instead, some genes in the *N. bombycis* genome were apparently derived from prokaryotes or viruses by horizontal gene transfer, similar to other microsporidian genomes [26,27]. Surprisingly, these prokaryote-transferred genes could complement some important metabolic pathways in *Nosema*, indicative of its essentiality over the course of *Nosema* evolution. The mobility of transposable elements has been shown to be associated with the frequency of horizontal gene transfer [40,41]. Although the *N. bombycis* genome is composed of ~38% repetivitive elements, only 55 genes were found to be horizontally transferred. Such observation indicated that a great number of transposons will not lead to higher rate of HGTs in *N. bombycis*. One explanation is that most transposons of *N. bombycis* have lost their activities such that the rate of HGTs is not enhanced*.* Alternatively, those repetitive elements do not possess the ability of capturing genes to facilitate the rate of HGTs.

The other major source of novel genetic materials in *N. bombycis* is the numerous paralogs by large-scale and small-scale duplication events. Some of them show evidence of accelerated changes through the relaxation of purifying selection, whereas others show evidence of positive selection. In either case, these paralogs seem to have provided raw materials for functional innovations as we showed in this study. Among them, the serine protease inhibitor family stands out as potential targets to study the higher infectious rates in *N. bombycis*.

## Methods

### Extraction of DNA, library construction, genome assembly, and annotation

About 1 × 10^9^ spores of *N. bombycis* CQ1, were isolated from infected silkworms in Chongqing. Using lysis buffer containing SDS and proteinase K, the *N. bombycis* genomic DNA was extracted from the germinated spores for each library construction. *N. antheraeae* YY isolate were collected from a farm in Henan of China and preserved in our lab, and then DNA was extracted with cetyl trimethylammonium bromide (CTAB) method. Detailed methods for extracting genomic DNAs can see the online supplementary materials (Additional file 16). After the library construction of plasmid and miniBAC, all sequence reads were obtained by Sanger and Illumina sequencing. To assemble the *N. bombycis* genome, the illumina reads were assembled by BGI’s *de novo* assembly software [42], which assembled unique and frequency repeat areas of genome. Next, we assembled further from the mixed data of illumina scaffolds and Sanger reads by using Phrap program.

To ensure the quality of gene annotation, we enriched the ESTs data by constructing two cDNA phage libraries and two Illumina cDNA libraries. 11,155 high quality reads were obtained by Sanger sequencing and 307,900 reads were obtained by illumina sequencing. Finally, 1517 unique ESTs were obtained with average length of 430 bp. Next, *N. bombycis* protein-coding genes were annotated using the following three different software: (1) Glimmer (version 3.0) with low eukaryote parameters [43], (2) GeneMarkS (version 4.6) with low eukaryote parameters [44], and (3) Augustus (version 2.0) with default parameters [45]. The details of library construction of plasmid, miniBAC, DNA and cDNA, as well as the protocol of genome assembly and annotations, for *N. bombycis* and *N. antheraeae* are provided as online supplementary materials. All annotated sequences of *N. bombycis* and *N. antheraeae* are deposited in Genbank as the following accession numbers: ACJZ01000001-ACJZ01003558.

### Identification horizontal gene transfer (HGT)

To examine the frequency of host-derived transposable elements, a phylogenetic analysis was conducted using the software RAxML [46] with the maximum likelihood (ML) algorithm. The amino acid replacement matrix, the WAG matrix, with gamma distribution was used to reconstruct the phylogenetic tree. Statistical support for nodes was estimated by using the bootstrapping method with 100 ML replicates. All other HGT genes of the *N.bombycis* genome were identified by using both the phylogenetic method and the Darkhorse methods [47]. For the phylogenetic method, all initial 4,458 *N. bombycis* genes were clustered to 3609 singletons at the level of ≥ 75% identity over ≥ 90% coverage for cluster members using BLASTCLUST program. A single randomly chosen representative of each cluster was used as a seed for BLASTP searches on nr database, the *Bombyx mori* genome database (http://silkworm.genomics.org.cn/). Sequences with E-value < 1e-5 and > 70% of the protein length) were aligned using clustal W. Bootstrap (100 replicates) consensus WAG model was made using RAxML to reconstruct Neighbor joining (NJ) trees. For the Darkhorse method, a filter threshold of 20% and two different self-definition keywords (*N. bombycis* and all species name of Microsporidia phylum) were used to eliminate the BLASTP matches by calculating the lineage probability index (LPI) of genes in the *N. bombycis* genome. Then, the potential horizontally transferred genes were retrieved.

### Identification of segmental and tandem duplications

To identify the segmental duplication, we performed all-against-all blast search with a single species to identify collinear regions within single genome as segmental duplicated blocks. A collinear region was defined as one where there are at least three homologous pairs with E value < 1E-6 and the distance between genes less than 5 kb. Segmental blocks were visualized using the software Circos-0.55 [48]. To plot duplicated blocks among *N. bombycis*, *N. antheraeae*, and *N. ceranae* genomes, we ordered the scaffolds as follows: 1) only the scaffolds that shared syntenic genes among these three species were included; 2) the scaffolds of *N. bombycis* were ranked from longest to shortest; 3) scaffolds of the other two species were arranged based on synteny to *N. bombycis*; 4) if *N. antheraeae* or *N. ceranae* scaffolds were syntenic to more than two scaffolds of *N. bombycis*, we define that scaffold order based on the longest scaffold of *N. bombycis*.

For the identification of tandem duplicates, we first classified gene family using the software MCL with E value < 1E-10, and then defined tandem duplicates as follows: 1) belonging to the same gene family, 2) being located within 5 kb each other, and 3) being separated by ≤ 3 non-homologous genes.

To time the age of paralogs, we first identified collinear regions between *N. bombycis* and *N. antheraeae*. Then, genes that lie in the collinear region were classified as orthologs between *N. bombycis* and *N. antheraeae*. Synonymous substitution rate (dS) of paralogs was estimated using the software Codeml in the package PAML 4 [49].

### Estimation of gene-wide selection and codon-based selection

The gene-wide selection and codon-based selection of genes in *N. bombycis* were analyzed following the procedure described in [29]. Briefly, clusters of homologous genes in *N. bombycis*, *N. antheraeae*, and *N. ceranae* identified by MCL (<1E-10) were grouped into four different categories: 1) clusters of orthologous genes (COGs) of 1:1:1 orthologous trios without any subsequent gene duplication in any species, 2) COGs of 1:1 *N. bombycis* and *N. antheraeae* gene pairs, 3) COGs of 1:1 *N. bombycis* and *N. ceranae* gene pairs, and 4) clusters of paralogous genes (CPGs) in *N. bombycis*. Prior to the estimation of dN/dS, those clusters with identity < 50% and an area covering <50% of the length of each sequence were filtered. Multiple alignments using MUSCLE [50] were then parsed to remove those poorly aligned regions using the Gblocks algorithm [51] with the following criteria: maximum number of contiguous non-conserved positions = 10 and minimum length of a block = 5. The amino acid alignment was back-translated into nucleotides sequence alignment. The gene-wide selection was then determined by calculating the median dN/dS value for each cluster. For the detection of codon-based positive selection, codon-based selection analysis was implemented using the Codeml program in the PAML package [49]. The site-specific model was used to detect positive selection in CPGs of *N. bombycis*. Two likelihood ratio tests were implemented: M1-M2 and M7-M8. M1 and M7 are the null models without positive selection, while M2 and M8 are the alternative models with positive selection. For each test, the first model (i.e., M1 or M7) is simpler than the second model (i.e., M2 or M8). To test if the second model fits better than the first model, twice difference of logarithm maximum likelihood estimates between the two compared models was compared against chi-square distribution with two degrees of freedom. Only those that showed posterior probability > 0.95 in the empirical Bayes method were considered as positively selected sites [52,53].

### RNA labeling and hybridization

RNA labeling and microarray hybridization was conducted by CapitalBio Corp (Beijing, China). Gene expression analysis was done based on the Affymetrix Silkworm Gene Chip kit in accordance following the manufacturer’s instruction (http://www.capitalbio.com). Briefly, after 5 × 10^4^ spores were fed to 3-instar larvae, total RNA was isolated from those 3-instar larvae at day 2, 4, 6, and 8. Then the extracted total RNA was reverse transcribed into cDNA. A dual-dye experiment was conducted. The uninfected cDNAs were labeled with dye Cy3 and infected cDNAs were labeled with dye Cy5. The labeled cDNA probes were dissolved in hybridization solution overnight at 42°C and then hybridized to the 23 k silkworm genome oligonucleotide chip (Capital Bio) that consists of 22,987 oligonucleotide 70-mer probes [54]. The signals were scanned with LuxScan 10KA scanner (CapitalBio corp).Three biological repeats were conducted at each time point.

## Competing interests

The author(s) declare that we have no competing interests.

## Authors’ contributions

Conceived and designed the experiments: ZYZ ZHX QYX JW CL ZZ NJH; Performed the experiments: GQP JSX TL CFL HDL LY XZ ZLW XQD MLT YHL; Analyzed the data: GQP JSX TL SLL GJZ SGL TL WF HX; Contributed reagents /materials/analysis tools: JHH ZL LPL JL LNG LLW MXL YJW; Wrote the paper: GQP ZYZ JSX QYX SLL PJK. All authors read and approved the final manuscript.

## Supplementary Material

Additional file 1 **Summary of reads data production in *N. bombycis.*** Click here for file

Additional file 2 **Statistics of genome assembly in *N. bombycis.*** Click here for file

Additional file 3 **The calculation of genomic size of *N. antheraeae *based on the frequency distribution of 15-mers depth of reads.** Click here for file

Additional file 4 **Gene ontology classification of three microsporidian *Nosema*.** Y axis: Log 10 (the proportion of gene numbers in certain sort occupied on the total Go-annotated gene numbers). Click here for file

Additional file 5 Comparison of average length of homologous gene among five different microsporidian species. Click here for file

Additional file 6 **List of main transposable elements among three *Nosema *species.** Click here for file

Additional file 7 The potential transposition of DNA sequences by Piggybac element. Click here for file

Additional file 8 Complete list of the annotation genes from genomes, and some lists of genetic position/names of identified element in text. Click here for file

Additional file 9 **Diagram showing the Piggybac transposon-mediated exogenous DNA sequence in the collinear region of *N. bombycis*.** TTAA indicates the recognition site of the Piggybac transposon. Click here for file

Additional file 10 **Summary of 55 horizontally transferred genes in the *N. bombycis *genome.** Click here for file

Additional file 11 **Figure showing phosphomevalonate kinase that horizontal transfer from bacteria integrates the mevalonate pathway of *N. bombycis*.** Click here for file

Additional file 12 **The location of segmental duplications in *Nosema bombycis* genome. ** Click here for file

Additional file 13 **Cumulative plot and statistics of the dN/dS values for CPG and COG genes in *N. bombycis*.** Click here for file

Additional file 14 The numbers of EST tags for positively selected CPG genes. Click here for file

Additional file 15 Table of parameter estimates for all positively selected CPGs. Click here for file

Additional file 16 Supplementary materials for library construction and genomic assembly. Click here for file
