# Supplementary material for: Comparative genomics of parasitic silkworm microsporidia reveal an association between genome expansion and host adaptation
Source: BMC Genomics. 2013 Mar 16;14:186. doi: 10.1186/1471-2164-14-186 (PMC3614468; doi:10.1186/1471-2164-14-186)
Supplement: Additional file 1 — Summary of reads data production in N. bombycis. [file 1471-2164-14-186-S1.docx]

**Table S1. Summary of reads data production in *N. bombycis***

|  | Insert size | Total length (Mb) | Sequence depth (X) | Ave. read length (bp) | Total reads | % reads used |
| --- | --- | --- | --- | --- | --- | --- |
| Mini-Bac ends | 10-30Kb | 6.56 | 0.42 | 480 | 13,673 | 86 |
| Plasmid ends | 2Kb | 104.24 | 6.62 | 451 | 231,293 | 83 |
|  | 8Kb | 2.13 | 0.14 | 455 | 4,694 | 59 |
| Solexa | 500bp | 443.36 | 28.2 | 75 | 2,955,733 | 89 |
| All |  | 556.29 | 35.38 | — | — | — |
