# Supplementary material for: Comparative genomics of parasitic silkworm microsporidia reveal an association between genome expansion and host adaptation
Source: BMC Genomics. 2013 Mar 16;14:186. doi: 10.1186/1471-2164-14-186 (PMC3614468; doi:10.1186/1471-2164-14-186)
Supplement: Additional file 2 — Statistics of genome assembly in N. bombycis. [file 1471-2164-14-186-S2.docx]

**Table S2. Statistics of genome assembly in *N. bombycis***

|  | N50 (bp) | number | Total length (bp) |
| --- | --- | --- | --- |
| contigs in scaffolds | 7,165 | 2,435 | 10,951,771 |
| contigs unassembled | 3,807 | 1,116 | 3,395,789 |
| scaffolds | 57,394 | 1,605 | 15,680,459 |
