# Supplementary figures and images for: Comparative genomics of parasitic silkworm microsporidia reveal an association between genome expansion and host adaptation
Source: BMC Genomics. 2013 Mar 16;14:186. doi: 10.1186/1471-2164-14-186 (PMC3614468; doi:10.1186/1471-2164-14-186)

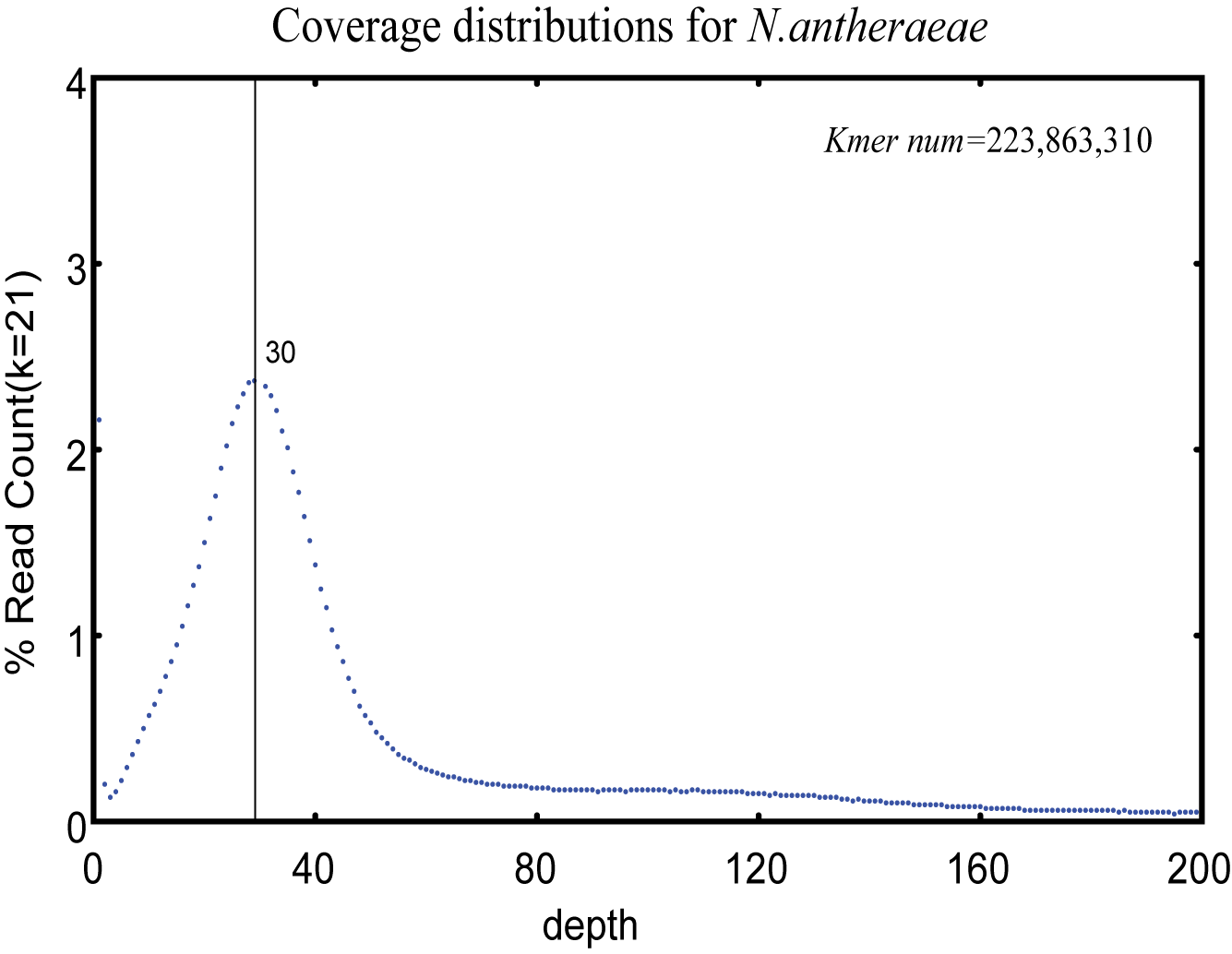

Supplement: Additional file 3 — The calculation of genomic size of N. antheraeae based on the frequency distribution of 15-mers depth of reads. [file 1471-2164-14-186-S3.tiff]

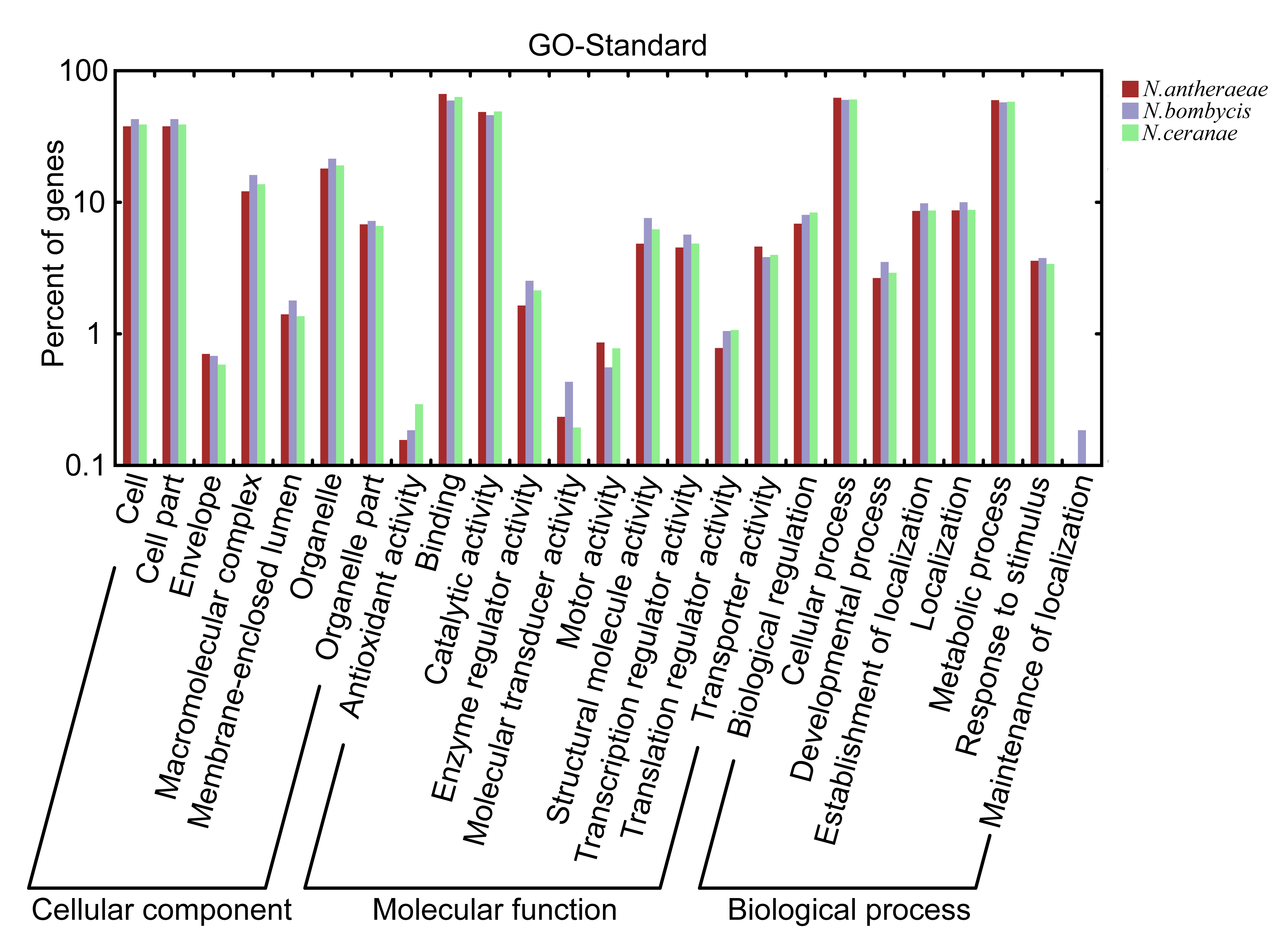

Supplement: Additional file 4 — Gene ontology classification of three microsporidian Nosema. Y axis: Log 10 (the proportion of gene numbers in certain sort occupied on the total Go-annotated gene numbers). [file 1471-2164-14-186-S4.tiff]

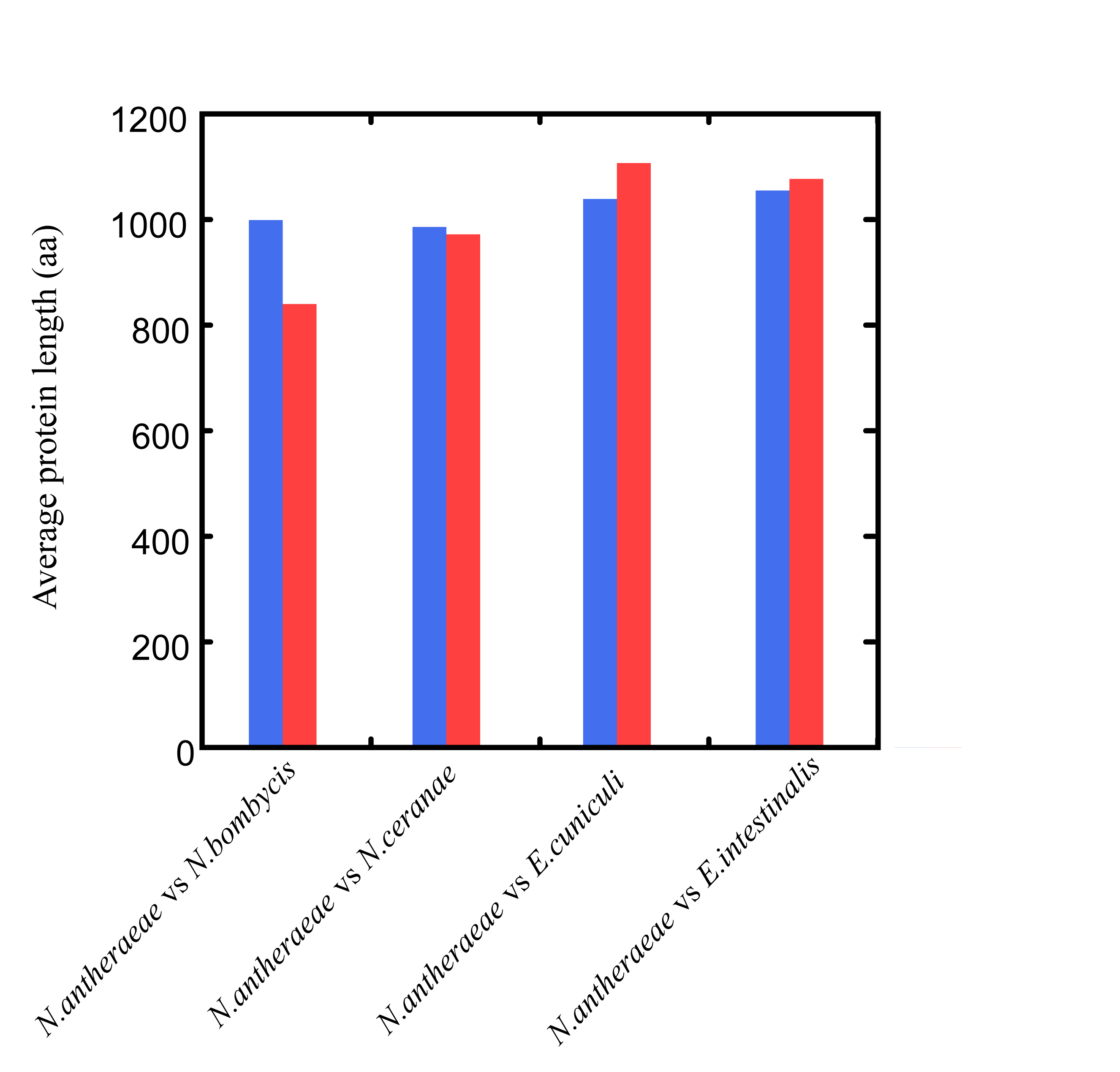

Supplement: Additional file 5 — Comparison of average length of homologous gene among five different microsporidian species. [file 1471-2164-14-186-S5.tiff]

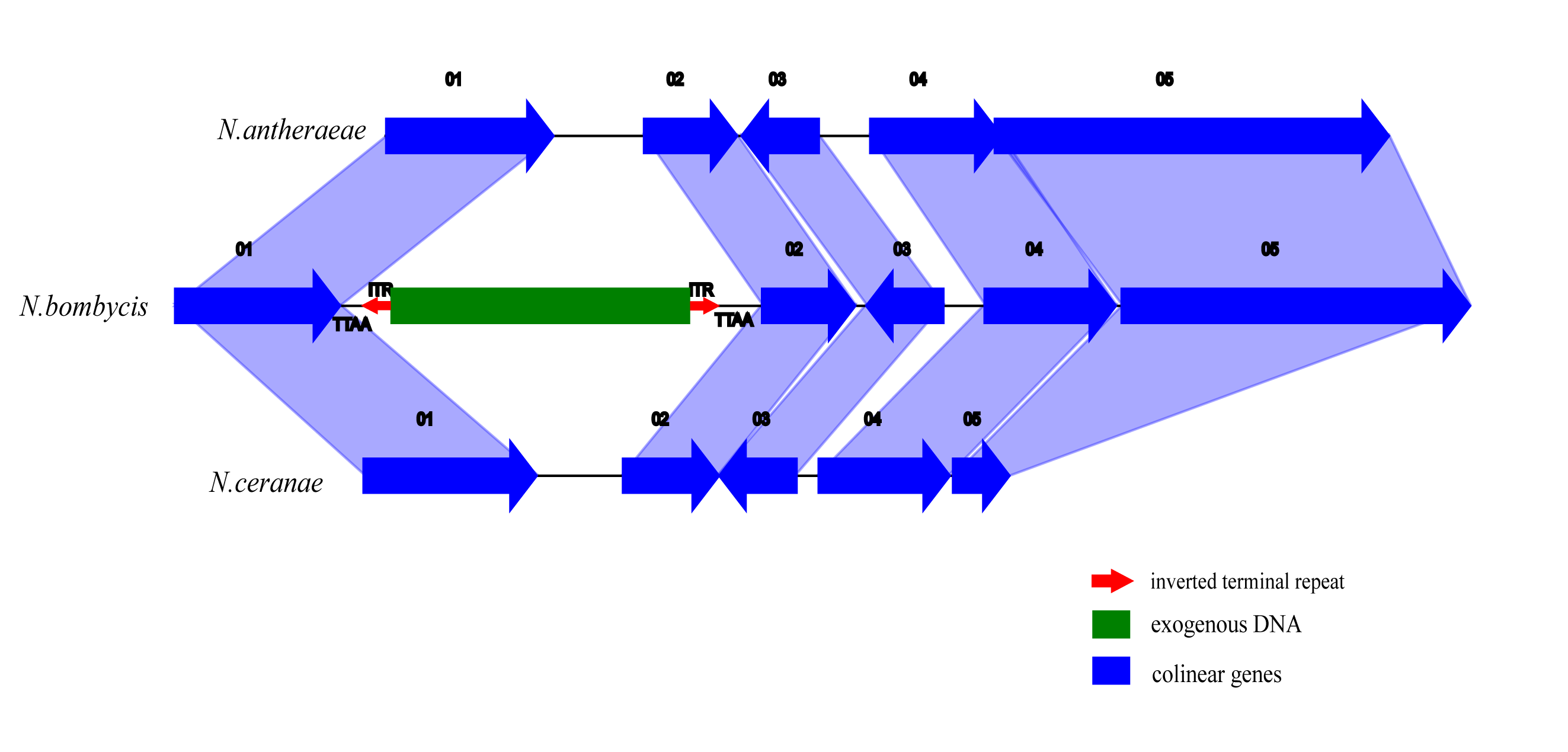

Supplement: Additional file 9 — Diagram showing the Piggybac transposon-mediated exogenous DNA sequence in the collinear region of N. bombycis. TTAA indicates the recognition site of the Piggybac transposon. [file 1471-2164-14-186-S9.tiff]

Supplemental Figure S7


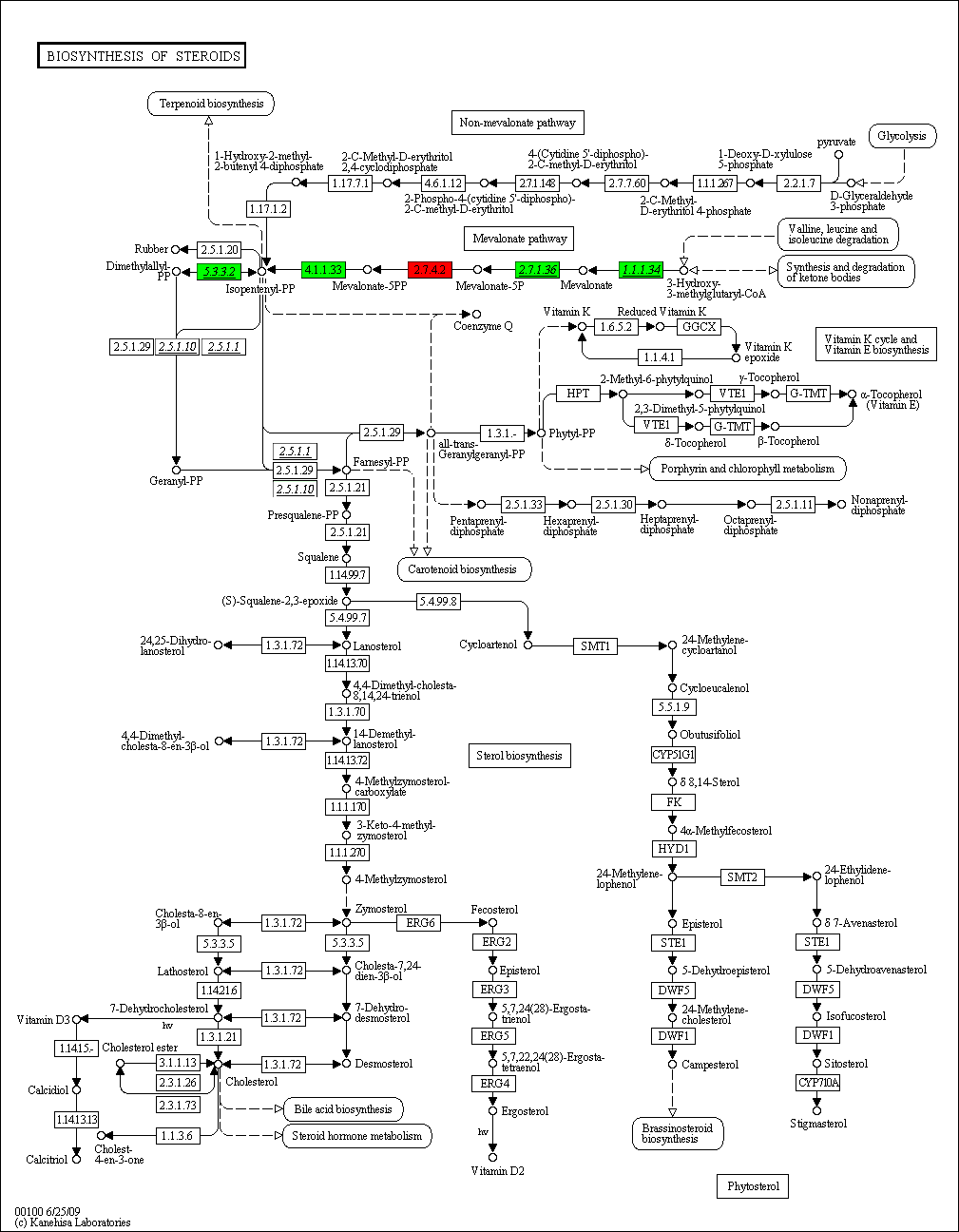

Supplement: Additional file 11 — Figure showing phosphomevalonate kinase that horizontal transfer from bacteria integrates the mevalonate pathway of N. bombycis. [file 1471-2164-14-186-S11.doc]

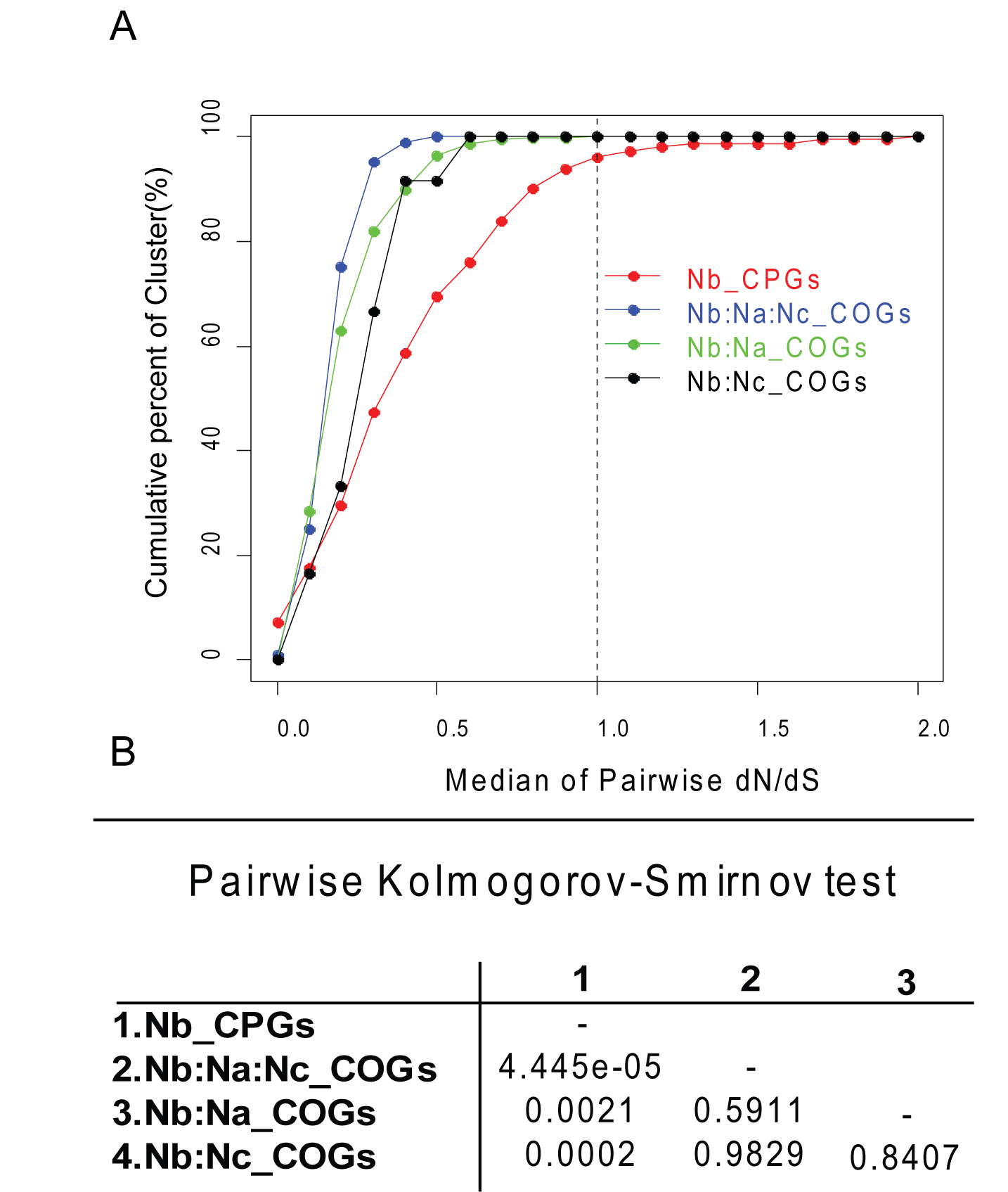

Supplement: Additional file 13 — Cumulative plot and statistics of the dN/dS values for CPG and COG genes in N. bombycis. [file 1471-2164-14-186-S13.tiff]
