# Supplementary material for: Comparative genomics of parasitic silkworm microsporidia reveal an association between genome expansion and host adaptation
Source: BMC Genomics. 2013 Mar 16;14:186. doi: 10.1186/1471-2164-14-186 (PMC3614468; doi:10.1186/1471-2164-14-186)
Supplement: Additional file 6 — List of main transposable elements among three Nosema species. [file 1471-2164-14-186-S6.docx]

**Table S3.** List of main transposable elements among three *Nosema* species.

|  | ***Nosema bombycis*** | | ***Nosema antheraeae*** | | ***Nosema ceranae*** | |
| --- | --- | --- | --- | --- | --- | --- |
| **Class** | **Copies** | **Total** | **Copies** | **Total/average** | **Copies** | **Total/average** |
| PiggyBac | 404 | 441876/1094 | 151 | 39998/265 | 0 | 0/0 |
| TcMar | 496 | 786733/1586 | 174 | 58983/339 | 137 | 44443/324 |
| Gypsy | 2248 | 577653/257 | 848 | 237726/280 | 226 | 63779/282 |
| R4 | 61 | 45069/739 | 50 | 17070/341 | 9 | 1299/144 |
