# Supplementary material for: Comparative genomics of parasitic silkworm microsporidia reveal an association between genome expansion and host adaptation
Source: BMC Genomics. 2013 Mar 16;14:186. doi: 10.1186/1471-2164-14-186 (PMC3614468; doi:10.1186/1471-2164-14-186)
Supplement: Additional file 7 — The potential transposition of DNA sequences by Piggybac element. [file 1471-2164-14-186-S7.docx]

**Table S4. The potential transposition of DNA sequences by piggyBac element.**

| **ITR type** | **Scaffold** | **Position** | **TSD** | **Annotation** |
| --- | --- | --- | --- | --- |
| NbPB1/BmPBLE12 | NBO_524 | 4316-5208 | + | **Unknown** |
| NbPB1/BmPBLE12 | NBO_359 | 7918-1121 | + | **Unknown** |
| NbPB1/BmPBLE12 | NBO_585 | 1246-2469 | + | **Unknown** |
| NbPB1/BmPBLE12 | NBO_487 | 19590-20302 | + | **Unknown** |
| NbPB1/BmPBLE12 | NBO_1089 | 1237-1990 | + | **Unknown** |
| NbPB1/BmPBLE12 | NBO_15 | 165422-166173 | + | **Unknown** |
| NbPB1/BmPBLE12 | NBO_22 | 225593-226470 | + | **Unknown** |
| NbPB1/BmPBLE12 | NBO_409 | 3964-4674 | + | **Unknown** |
| NbPB1/BmPBLE12 | NBO_409 | 3964-5402 | + | **Unknown** |
| NbPB1/BmPBLE12 | NBO_51 | 55365-56121 | + | **Unknown** |
| NbPB1/BmPBLE12 | NBO_8 | 302839-304082 | + | **Unknown** |
| NbPB1/BmPBLE12 | NBO_1510 | 345-1246 | + | **Unknown** |
| NbPB1/BmPBLE12 | NBO_594 | 3621-4375 | + | **Unknown** |
| NbPB1/BmPBLE12 | NBO_36 | 27927-28680 | + | **Unknown** |
| NbPB1/BmPBLE12 | NBO_986 | 2192-3088 | + | **Unknown** |
| NbPB1/BmPBLE12 | NBO_12 | 272016-272769 | + | **Unknown** |
| NbPB1/BmPBLE12 | NBO_12 | 328257-329010 | + | **Unknown** |
