# Supplementary material for: Comparative genomics of parasitic silkworm microsporidia reveal an association between genome expansion and host adaptation
Source: BMC Genomics. 2013 Mar 16;14:186. doi: 10.1186/1471-2164-14-186 (PMC3614468; doi:10.1186/1471-2164-14-186)
Supplement: Additional file 16 — Supplementary materials for library construction and genomic assembly. [file 1471-2164-14-186-S16.doc]

**Supplementary materials**

**Construction of *N. bombycis* plasmid libraries.** The pellets of 10^9^ purified spores were suspended in 400μL germination buffer (0.1M Na2CO3, 0.15M NaCl, pH10.8), then put on ice for 30min. 9.25μL 3M NaAc (pH4.8) were added to ajust the pH to 8.0, ice bath for 15min. After that, 136μL 2% SDS were added into the spore solution, then put on ice for another 30min; then, added the proteinase K to 1mg/mL, water bathed at 50℃ 4 hours or overnight. An equal volume TE-saturated phenol (pH8.0) was added into the samples to extract the DNA, collect the supernatant for another extraction with Chloroform: isoamyl alcohol (24:1), after centrifuge the water phase was collected mildly. Then 0.1 volume 3M NaAc and two volume ethanol (conserved in -20℃) were added to precipitate the DNA at -20℃ for 30min. and then centrifuge at 13000rpm for 10min, remove the supernatant cautiously, 70% ethanol was used to desalt the genomic DNA. Finally the DNA samples were dissolved in 1X TE for Genomic library construction. Qualified genomic DNA were hydrosheared into pieces, and 1.5-2.5 Kb and 7-8 Kb fragments were collected and linked with pUC18 respectively, then transform the plasmid into E. coli JM109. Totally, 239,987 Sanger reads were obtained and average length per read is 453 bp.

**Construction of *N. bombycis* miniBAC library.** Plugs for PFGE analyses were prepared as follows: the purified spores (~10^9^ spores/plug) were suspended by 1.4% low melting point agarose, and transferred into the plug mold on ice, then the solidified agarose plugs were incubated in solutionI (EDTA, 0.1M; SDS, 5%; DTT, 0.1M) at 50℃ for 2 h, before treatment with solutionⅡ (EDTA, 0.5M; SLS, 0.5%; Proteinase K, 1.5mg/mL) at 50℃ for 48 h. The plugs were incompletely digested with HindIII before running PFGE. And 10-30kb fragments were collected and a miniBAC library was constructed with Copy Control TM BAC Cloning Kits from EPICENTRE Biotechnologies. 13,673 Bac-ends were sequenced with an average length 480.0 bp.

**Construction of *N. bombycis* Illumina DNA library.** Illumina library preparation followed the manufacturer’s instructions (Illumina). Briefly, 2–5 ug of genomic DNA in 50ul TE buffer were fragmented by nebulization with compressed nitrogen gas at 32psi for 9 minutes. Nebulization generated double-stranded DNA fragments with blunt ends or with 3’ or 5’ overhangs. The overhangs were converted to blunt ends using T4 DNA polymerase and Klenow polymerase, after which an “A” base was added to the ends of double-stranded DNA using Klenow exo- (3’ to 5’ exominus). Next, DNA adaptors (Illumina) with a single “T” base overhang at the 3’ end were ligated to the above products. These products were then separated on a 2% agarose gel, excised from the gel at a position between 150 and 250 bp, and purified (Qiagen Gel Extraction Kit). The adapter-modified DNA fragments were enriched by PCR with PCR primers 1.1 and 2.1 (Illumina). Separate 8-, 10-, 12-, 15-, and 18-cycle reactions were used for sequencing. The concentration of the libraries was measured by absorbance at 260nm. The template DNA fragments of the constructed libraries were hybridized to the surface of flow cells and amplified to form clusters. After dsDNA was denatured to ssDNA and non-specific sites were blocked, genomic DNA sequencing primers were hybridized for DNA sequencing initiation. In brief, cluster generation was performed on the Illumina cluster station, and the basic workflow (based on the standard Illumina protocol) was as follows: Template hybridization, isothermal amplification, linearization, blocking, and denaturization and hybridization of the sequencing primers. The fluorescent images were converted to sequence using the Illumina base-calling pipeline (SolexaPipeline-0.2.2.6).

**Construction of *N. bombycis* cDNA library.** Two cDNA phage libraries were constructed with purified spores and heavily *N. bombycis*-infected silk glands respectively. The libraries were constructed following the protocol of the Creator SMART cDNA Library Construction Kit (Clonetech). 11,155 reads were acquired by Sanger sequencing method and 1,517 unique ESTs were obtained with average length is 391bp.

**Construct the *N. bombycis* Illumina cDNA library.** The heavy infected silk glands and midguts of silkworm by *N. bombycis* were collected and preserved in liquid nitrogen. Samples were grounded into powder in liquid nitrogen and total RNA was extracted with Trizol Reagent (Invitrogen). The quality of total RNA was checked by UV spectrometry and electrophoresis. The qualified total RNA was used for further test. The mRNA is fragmented into small pieces using RNA fragmentation buffer (Ambion) under elevated temperature of 70 °C for exactly 5minutes. Immediately, the fragmented RNA was precipitated using 100% ethanol combined with glycogen (Ambion) and acetate sodium (Novagen). 1µg random hexa-primer (IDT) was introduced to first strand cDNA synthesis reaction, also mixed with 200U of Superscript II reverse transcriptase (Invitrogen), 40U of RNaseOUT™, 0.1M DTT, and 10mM dNTPs (NEB) in a total volume of 20 µl; this reaction mix was incubated at 25°C for 10 minutes and 42°C for 30 minutes, followed by inactivate the reaction at 70°C for 15 minutes. Then the second strand was synthesized in a reaction mix containing 10mM dNTPs (NEB), 50U of E. coli DNA PolymeraseⅠ (Invitrogen) and 4U of E. coli RNase H (Invitrogen) in a total volume of 100 µl; this reaction mix was incubated at 16°C for 2 hours. The resulting double strands cDNA was purified using the Qiaquick PCR purification kit (Qiagen). In order to convert the overhangs into blunt ends, cDNA was treated by 15U of T4 DNA polymerase (NEB) and 5U of Klenow polymerase (NEB), meanwhile 30U of T4 polynucleotide kinase (NEB) was used to phosphorylate the 5’ ends in the presence of 10mM ATP; this reaction mix was incubated at 20°C for 30 minutes. A 3’ overhang was generated using 3’-5’ exonuclease deficient Klenow fragment (NEB) in the presence of 1 mM dATP (NEB) by incubating at 37°C for 30 minutes. Then the cDNA was ligated to Illumina PE adapter oligo mix. In two times of gel electrophoresis, cDNA fragment was selected as size-range around 200bp. Before cluster generation on flow cell for Illumina sequencing, we assessed the quality of RNA-seq library by Bioanalyzr 2100 (Agilent) and its concerntration using Taqman real-time PCR. Library material was loaded onto one lane of the flow cell at regularly 2~8 pM concentration. Finally, paired-ends sequencing was performed by running 45 cycles using Illumina reversible-terminator chemistry on the Illumina Genome Analyser.

**Construction of *N. antheraeae* Illumina DNA library.** Samples of *N. antheraeae* were collected from silkworm farms in Henan Province, China. After collection, the isolation of Nosema followed the protocol described in Ref. The isolated strain YY was then preserved in -80°C freezer. Total DNA from a tissue that comprises of approximately 1010 Nosema spores was extracted using the CTAB method. 20 μg of total DNA was subsequently used to construct 500 bp gene clone library followed the manufacturer’s instructions for a 100-bp paired-end sequencing (Illumina).

**Genome assembly**

By sequencing *N. bombycis*, we got about 6.7X WGS plasmid Sanger reads (with 2Kb inserts), 0.4X miniBAC ends (~10X physical coverage) and ~28X illumina reads (Table S1), from which the genome was assembled. First, the illumina reads were assembled by BGI’s de novo assembly software (Li, Zhu et al. 2010),which assembled unique and frequency repeat areas of genome. We next mixed scaffolds from illumina assembly and Sanger reads data to assemble by using Phrap program. We blast these Sanger reads and illumina contigs against GenBank non-redundant nucleotides (nt) database to filter polluted DNA (BLASTN with 1e-5). Then we analyzed the distribution of 20-mer depth and masked out high-depth reads before Phrap to prevent mis-assembly caused by repeat. Because of the superabundance and complex component of repetitive sequences, the genome sequence of *N. bombycis* is highly fragmented. Thus this has greatly prevented the investigation of *N. bombycis* genome architecture. We closed gaps between adjacent contigs in a scaffold by extending reads whose 3’ end is oriented toward the intervening gap. Finally, we closed 114 intra-scaffold gaps in the *N. bombycis* assembly. Other post-assembly procedures utilized in the reported genome of Trichomonas vaginalis, a species with high repetitive components similar with *N. bombycis*, were also used to improve the assembly quality. However, little improvement was achieved via these procedures (data not shown). The final assembly contains 3,551 contigs totaling 14.3 Mb, of which 2,435 (11.0 Mb) have been placed into scaffolds (Table S2). The average size of contigs is about 4.0 Kb. The N50 of scaffolds is about 57.4 Kb, and the final assembly dataset with gaps is totally 15,680,459 bp in size.

By sequencing *N. antheraeae*, 900Mb raw reads were obtained. To remove any ambiguous reads，we filtered out 1) reads with non-determined bases (i.e., N) greater than 10 %, 2) reads with low quality Q20 score more than 40 bp, 3) reads with poly A structures. We also remove the contaminations of adaptors by only allowing the 24 bp overlapping region between reads and adaptors. After these filtration steps, 657 Mb raw reads were kept for assembly. The assembly was performed using the software SOAPdenovo.

**Annotation of protein-coding region, transposable elements, and regulatory motifs**

We identified protein-coding genes from 3 types of sources: (1) de novo prediction, include Glimmer (version 3.0) with low eukaryote parameters , GeneMarkS (version 4.6) with low eukaryote parameters, and Augustus (version 2.0) with default parameters. Glimmer used the following parameters: a minimum length of 100 amino acids, a maximum overlap of 50 bp, and a threshold score of 30. While GeneMarkS used a self-training algorithm and Augustus used the yeast-trained models. (2) Compared our assembled genome with published microsporidian protein coding genes using the software tBLASTn（E value <1E-5）(3) EST/unigene mapping of *N. bombycis*, map the EST sequences to *N. bombycis* genome by BLAT, and assembled on genomic locus by PASA. We finally combined all the gene sets into a single reference gene set, so that researchers could further utilize for consistent and comparable analyses.

Repetitive sequences of *N. bombycis* were predicted using four de novo softwares, ReAS, PILER-DF, RepeatScout and LTR_Finder. The input for ReAS is 1X coverage of Sanger reads, while the input for other softwares is the assembled scaffolds. We required the TE length to be larger than 100 bp and Gap “N” less than 5%, and combined TE predictions from the four softwares to form a raw TE library. We next filtered the redundancy by performing all-versus-all BLASTN for TE sequences with E-value 1e-10, and filtered the shorter one when two TEs aligned with identity>=80% and coverage>=80% and matching length>=100bp. We also filtered the high-copy number protein-coding genes, by BLASTX the TE sequences against Swissprot at E-value 1e-4, with cutoffs of identity>=30% and coverage>=30% and matching length>=30aa. At last, we got a pure *N. bombycis* TE library, which was followed by manual check. We assigned types to de novo TE families by aligning the TE sequences to various known TE DNA and protein databases, and then combined these results based on a priority order of the approaches: (1) BLASTN to Repbase (update 14.12) TEs; (2) BLASTX to TE proteins; (3) BLASTX to Swissprot proteins; (4) TBLASTX to Repbase TEs. The priority order is: (1) > (2) > (3) > (4). If one TE is annotated by a higher priority approach, then the lower approaches will be neglected. For DNA level aligning, we require cutoffs of E-value<=1e-5, coverage>=30% and match length >= 80 bp. For protein level aligning, all the other approaches, the cutoffs are E-value <= 1e-4, identity>=30%, coverage>=30% and match length>=30 aa. For the LTR_Finder predicted TEs, they are classified as "LTR" if they haven't a significant hit to known TE and can't assign a more detailed type. The automatic type assignment was further checked and corrected by manual check. The classification of transposable elements was based on ref . And then, to compare different types and numbers of transposable elements among *N. bombycis*, *N. antheraeae* and N.ceranae, RepeatMasker software was used to search *N. antheraeae* assembled genomic sequence and N.ceranae published genome against the data of *N. bombycis* repetivitive elements we identified above.

Reference

1. Xu J, Zhou Z (2010) Improving phylogenetic inference of microsporidian *Nosema* *antheraeae* among Nosema species with RPB1, α-and β-tubulin sequences. African Journal of Biotechnology 9: 7900-7904.

2. Carlton JM, Hirt RP, Silva JC, Delcher AL, Schatz M, et al. (2007) Draft genome sequence of the sexually transmitted pathogen *Trichomonas vaginalis*. Science 315: 207.

3. Li R, Zhu H, Ruan J, Qian W, Fang X, et al. (2010) De novo assembly of human genomes with massively parallel short read sequencing. Genome research 20: 265.

4. Delcher AL, Harmon D, Kasif S, White O, Salzberg SL (1999) Improved microbial gene identification with GLIMMER. Nucleic acids research 27: 4636.

5. Lomsadze A, Ter-Hovhannisyan V, Chernoff YO, Borodovsky M (2005) Gene identification in novel eukaryotic genomes by self-training algorithm. Nucleic acids research 33: 6494.

6. Stanke M, Diekhans M, Baertsch R, Haussler D (2008) Using native and syntenically mapped cDNA alignments to improve de novo gene finding. Bioinformatics 24: 637.

7. Li R, Ye J, Li S, Wang J, Han Y, et al. (2005) ReAS: recovery of ancestral sequences for transposable elements from the unassembled reads of a whole genome shotgun. PLoS computational biology 1: e43.

8. Edgar RC, Myers EW (2005) PILER: identification and classification of genomic repeats. Bioinformatics 21: i152.

9. Price AL, Jones NC, Pevzner PA (2005) De novo identification of repeat families in large genomes. Bioinformatics 21: i351.

10. Xu Z, Wang H (2007) LTR_FINDER: an efficient tool for the prediction of full-length LTR retrotransposons. Nucleic acids research 35: W265.

11. Altschul SF, Madden TL, Sch ffer AA, Zhang J, Zhang Z, et al. (1997) Gapped BLAST and PSI-BLAST: a new generation of protein database search programs. Nucleic acids research 25: 3389.

12. Jurka J, Kapitonov V, Pavlicek A, Klonowski P, Kohany O, et al. (2005) Repbase Update, a database of eukaryotic repetitive elements. Cytogenetic and genome research 110: 462-467.

13. Wicker T, Sabot F, Hua-Van A, Bennetzen JL, Capy P, et al. (2007) A unified classification system for eukaryotic transposable elements. Nature Reviews Genetics 8: 973-982.
